# Force-limited distance-measurable nerve root retractor plus intraoperative neurophysiological monitoring reduces L5 radiculitis in posterior lumbar interbody fusion

**DOI:** 10.1186/s13018-025-06429-0

**Published:** 2025-11-14

**Authors:** Min Wang, Zhiqing Chen, Jibin Ma, Yufei Yuan, Jun Miao, Wangqiang Wen, Bangquan Liao

**Affiliations:** 1 Clinical School, College of Orthopedics, Tianjin Medical University, No. 22 Qixiangtai Road, Heping District, Tianjin, China; 2 Department of Orthopedics (Spine Division), Beichen Hospital Affiliated to Nankai University, No. 7 Beiyi Road, Beichen District, Tianjin, China; 3 Clinical College of Orthopedics, Tianjin Medical University, 406 Jiefang South Road, Hexi District, Tianjin, China; 4 Department of Orthopedics, The Second People’s Hospital of Changzhi, No. 83 Peace West Street, Luzhou District, Changzhi, Shanxi Province China; 5 The Orthopedic Surgery Department of Handan Central Hospital, 15 Zhonghua South Street, Handan, 056001 Hebei China; 6 Department of Spine Surgery, Tianjin Hospital, Tianjin University, Jiefangnanlu 406, Hexi District, Tianjin, China; 7 The First Affiliated Hospital of Hainan Medical University (Hainan Province Clinical Medical Center, National Clinical Key Specialty Construction Department), Haikou, Hainan China; 8 School of Physical science and technology, Tiangong University, Tianjin, China

**Keywords:** Lumbar spinal stenosis, Posterior lumbar interbody fusion, Nerve root protection, Intraoperative neurophysiological monitoring, Force-limited nerve root retractor, L5 radiculitis

## Abstract

**Objective:**

To evaluate the incidence of L5 radiculitis in patients undergoing posterior lumbar interbody fusion (PLIF) using a force-limited distance-measurable nerve root retractor (FLDM-NRR) combined with intraoperative neurophysiological monitoring (IONM), compared with conventional nerve root retraction.

**Methods:**

This retrospective cohort study examined 234 patients with L4-L5 lumbar spinal stenosis (LSS) undergoing single-level PLIF from January 2022 to March 2024. Patients were categorized into the FLDM-NRR plus IONM group (*n* = 96) with force limitation ≤ 3.5 N, and the conventional nerve root retraction group (*n* = 138). The primary outcome was 3-month L5 radiculitis; Secondary outcomes included 1-year persistent neurological impairment, Japanese Orthopedic Association (JOA) scores, Oswestry Disability Index (ODI), Visual Analog Scale (VAS) pain scores, and achievement of minimal clinically important difference (MCID). Multivariable logistic regression, mixed-effects models, and hierarchical testing were applied.

**Results:**

FLDM-NRR with IONM reduced the 3-month L5 radiculitis incidence (7.29% vs 18.12%, adjusted OR = 0.31, 95% CI 0.11–0.78; *P* = 0.017; absolute risk reduction (ARR) = 10.83%; number needed to treat (NNT) = 9), with greatest protection in the first postoperative week (3.13% vs 13.04%, *P* = 0.020). No significant difference was observed in 1-year persistent neurological impairment (3.13% vs 7.25% *P* = 0.200). The FLDM-NRR group showed superior ODI (18.43 ± 7.85% vs 21.17 ± 8.42%, *P* = 0.018) and higher MCID rates in VAS leg pain (95.8% vs 93.5%, *P* = 0.028). IONM demonstrated 85.71% sensitivity and 96.63% specificity in predicting postoperative complications. The FLDM-NRR system consistently upheld the predetermined force threshold in all instances without any device-related problems.

**Conclusion:**

FLDM-NRR combined with IONM significantly reduces early L5 radiculitis after PLIF and provides short-term functional benefits. Long-term neuroprotection remains unproven and requires validation in prospective randomized trials.

**Supplementary Information:**

The online version contains supplementary material available at 10.1186/s13018-025-06429-0.

## Introduction

Lumbar spinal stenosis (LSS) represents a common degenerative spinal condition widespread among the elderly, with clinical incidence rates between 11% and 39% [1]. This syndrome is defined by the constriction of the spinal canal, lateral recess, or neural foramina, leading to neurogenic claudication, radicular discomfort, and increasing functional deterioration [2]. When conservative therapy is ineffective, posterior lumbar interbody fusion (PLIF) serves as the conventional surgical intervention for symptomatic LSS [3, 4].

Neurological issues associated with surgery considerably affect patient outcomes in PLIF surgeries. Research indicates that PLIF has a nerve root damage risk of up to 7.8%, significantly above the 2.0% rate linked to transforaminal lumbar interbody fusion (TLIF) [5, 6]. Endoscopic techniques have further been shown to reduce nerve-related complications via precise nerve root protection [7, 8]. The L5 nerve root is particularly susceptible to injury during surgical procedures, primarily due to its elongated course beneath the neural foramen and close proximity to adjacent tissues [9–11]. Injury may arise from traction, compression, or severe retraction during fusion operations. Traditional retraction methods rely on the surgeon’s expertise and tactile perception, lacking objective quantitative benchmarks and resulting in inter-operator variability.

However, Intraoperative neurophysiological monitoring (IONM) offers real-time neurological evaluation using multimodal approaches and is the prevailing standard for neuroprotection in spine surgery [12, 13]. IONM demonstrates specific limitations in identifying mechanical traction injuries, since electrophysiological alterations may trail after the actual nerve damage [14]. The time delay, exacerbated by IONM’s vulnerability to anesthetic and hemodynamic disruptions, may permit irreparable damage to transpire prior to detection [15]. These constraints have stimulated interest in supplementary mechanical protection techniques.

The force-limited, distance-measurable nerve root retractor (FLDM-NRR) was developed based on biomechanical principles to limit nerve root retraction force to *≤* 3.5 N while enabling real-time measurement of retraction displacement. Biomechanical research indicates that neural tissues possess defined tolerance levels to mechanical stress, beyond which permanent damage ensues [16–19]. In 2023, our hospital adopted FLDM-NRR technology with IONM as a component of our neuroprotection strategy for PLIF surgeries.

Clinical data concerning the efficacy of force-controlled nerve root protection is limited. Previous studies have largely focused on biomechanical analyses and small case series, and large-scale clinical evaluations comparing outcomes between force-limited and traditional retraction techniques remain insufficient. The appropriate combination of mechanical force control with electrophysiological monitoring has not been thoroughly evaluated in clinical practice.

The implementation of FLDM-NRR technology at our institution enabled a comparison of results with our prior standard practice using traditional retraction procedures. This retrospective cohort research evaluates the clinical efficacy of FLDM-NRR combined with IONM, compared with conventional retraction, in reducing the risk of postoperative L5 radiculitis in patients undergoing single-level L4-L5 PLIF for LSS. It was hypothesized that force-controlled nerve root retraction would reduce the incidence of postoperative L5 radiculitis while maintaining surgical efficacy. Ultimately, this study aims to provide clinical evidence supporting the application of standardized mechanical neuroprotective strategies in spinal surgery.

## Methods

### Study design and population

This retrospective cohort study analyzed clinical data from patients who underwent single-level L4-L5 PLIF at our spinal surgery department between January 2022 and March 2024. The protocol was approved by our institutional review board (IRB), and all procedures complied with the Declaration of Helsinki and relevant international medical ethical standards. For this study, a total of 234 patients were included who underwent single-level L4-L5 PLIF and met the inclusion criteria throughout the study period.

*Inclusion criteria*: aged 40–80 years with single-level L4-L5 moderate to severe LSS, as confirmed by magnetic resonance imaging (MRI). The severity of stenosis was evaluated using the Lee grading criteria [20], with a dural sac cross-sectional area of ≤ 100 mm². The supplementary inclusion criteria included: (1) symptomatic neurogenic claudication or radicular pain refractory to conservative treatment for ≥ 3 months; (2) American Society of Anesthesiologists (ASA) classification ≤ II; (3) undergoing PLIF surgery; (4) clinical data being accessible during preoperative, postoperative, and follow-up periods.

#### Exclusion criteria

(1) a previous lumbar surgery at the same level; (2) preoperative presence of spinal infection, neoplasm, or acute fracture; (3) significant comorbidities that impair neurological evaluation (severe diabetic peripheral neuropathy, myelopathy, severe peripheral vascular disease); (4) inadequate clinical documentation or missing follow-up information.

**Fig. 1:**
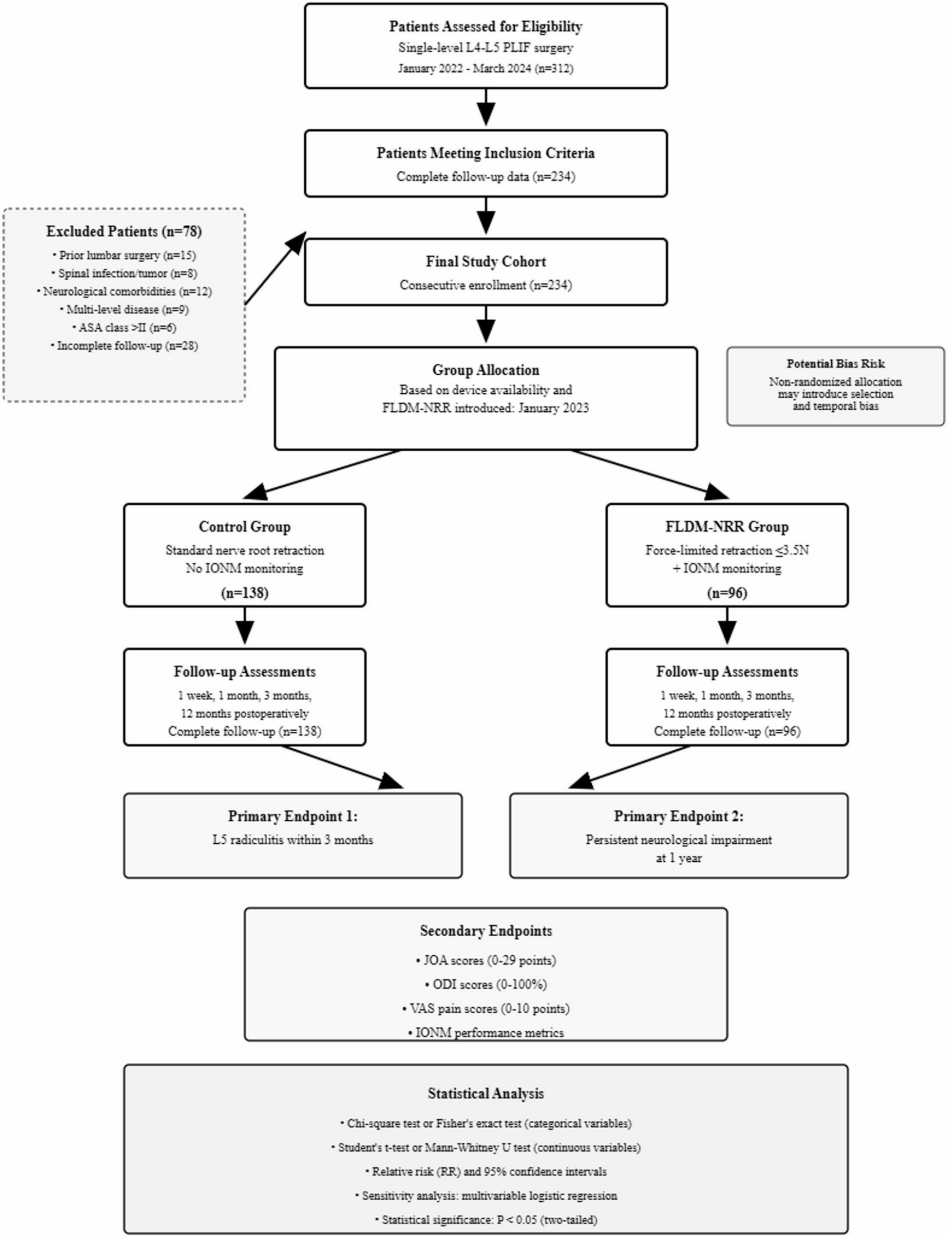
CONSORT flow diagram for patient selection and allocation

**Fig. 2:**
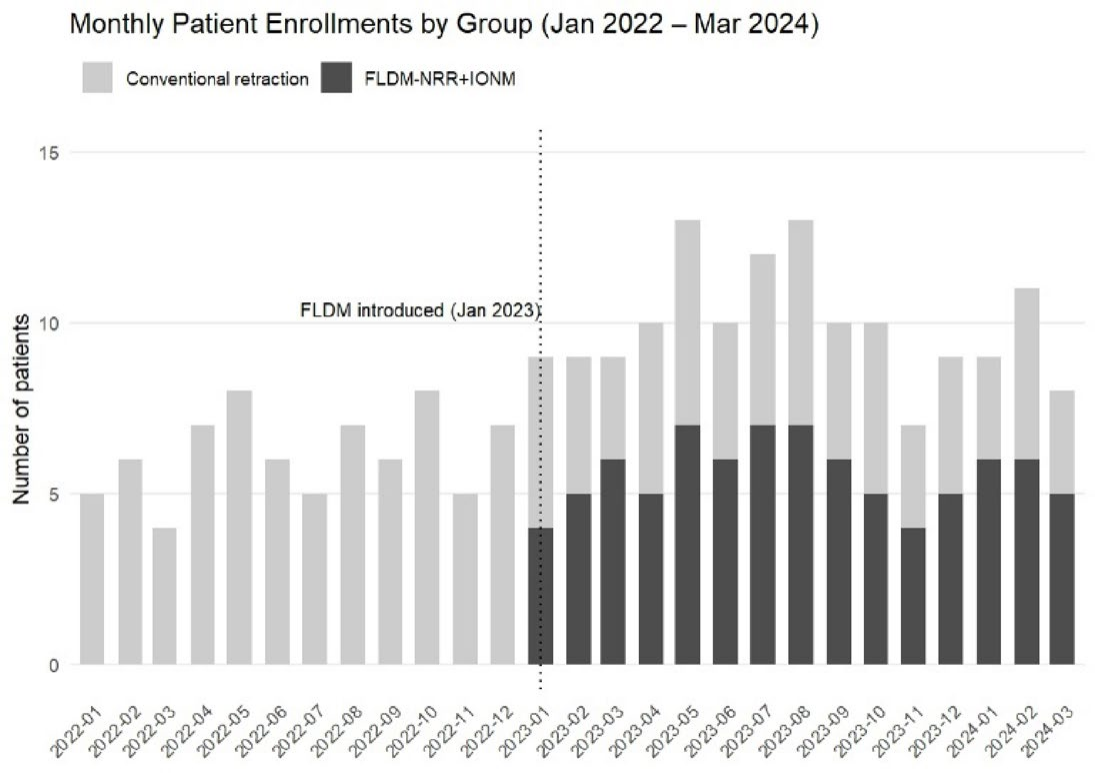
Monthly patient enrollments by retraction strategy (Jan 2022–Mar 2024). Conventional retraction (light gray) and FLDM-NRR + IONM (dark gray). Vertical dotted line = institutional FLDM-NRR introduction (Jan 2023). Both strategies used concurrently (Jan 2023–Mar 2024)

Patients were allocated to neuroprotection strategies based on objective FLDM-NRR device availability following its institutional introduction in January 2023. Device accessibility was determined by sterilization cycles and maintenance schedules, with no subjective selection criteria influencing group assignment. Clinical data showed that a total of 234 patients were enrolled: 96 in the FLDM-NRR with IONM group and 138 in the traditional nerve root retraction group. (Fig. 1) Due to intermittent device availability, both strategies were used concurrently from January 2023 to March 2024, ensuring contemporaneous recruitment. A calendar timeline of monthly enrollments is shown (Fig. 2), with detailed counts provided in Supplementary Table Sx.

### Data extraction and standardization procedures

Data extraction was performed by independent research assistants who were blinded to group allocations (FLDM-NRR or conventional retraction) to prevent bias. Only essential information for analysis was provided, minimizing observer bias.

Inter-rater agreement was assessed using Cohen’s Kappa statistic, showing high consistency with Kappa values of 0.85 for pain and 0.82 for neurological evaluations, indicating excellent reliability.

Postoperative visit schedules and electronic medical record (EMR) templates were standardized across both groups, ensuring consistent data recording. Pain and neurological assessments followed a uniform protocol using the Japanese Orthopedic Association (JOA) score, Oswestry Disability Index (ODI), and Visual Analog Scale (VAS) for pain, applied at all follow-up visits (1 week, 1 month, 3 months, and 12 months) to minimize detection bias.

Retrospective data extraction employed dual independent extraction with cross-verification, keeping key variable missing rates < 5%. Of 312 initially assessed medical records, 50 were excluded based on inclusion/exclusion criteria, leaving 262 patients eligible for 12-month follow-up. Among these, 28 were lost to follow-up, and 234 completed follow-ups, yielding an 89.3% (234/262) 12-month follow-up data completeness rate—well above the acceptable threshold and far below the 15% clinical follow-up loss threshold and meeting retrospective cohort study data completeness requirements.

### Surgical technique and monitoring

All surgeries were conducted by the same spinal surgery team using a standardized posterior midline approach. The PLIF procedure involved pedicle screw insertion, laminectomy, and decompression of the L5 nerve root. Nerve root retraction was performed at the shoulder region of the L5 nerve root, -proximal to the dural sleeve transition zone, where the nerve root exits the thecal sac.

**Fig. 3:**
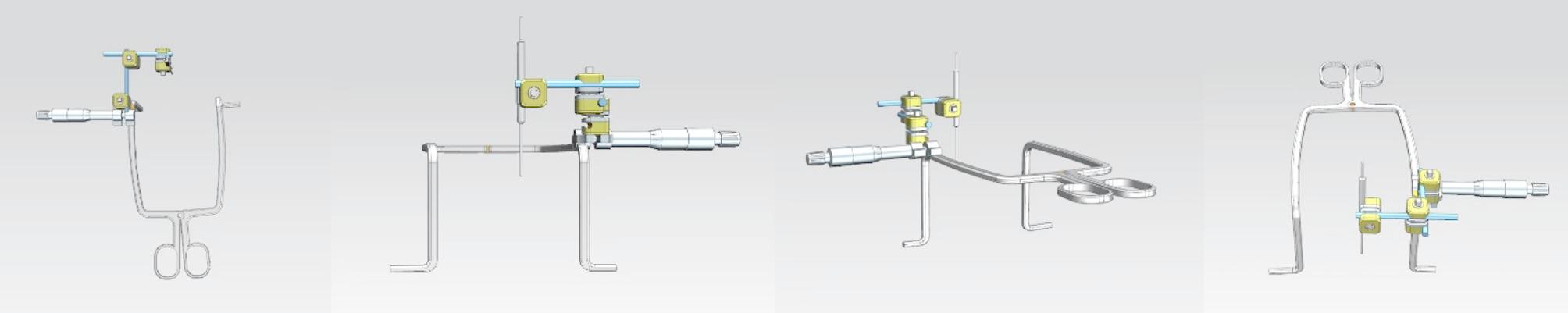
Design schematic of the FLDM-NRR device

**Fig. 4:**
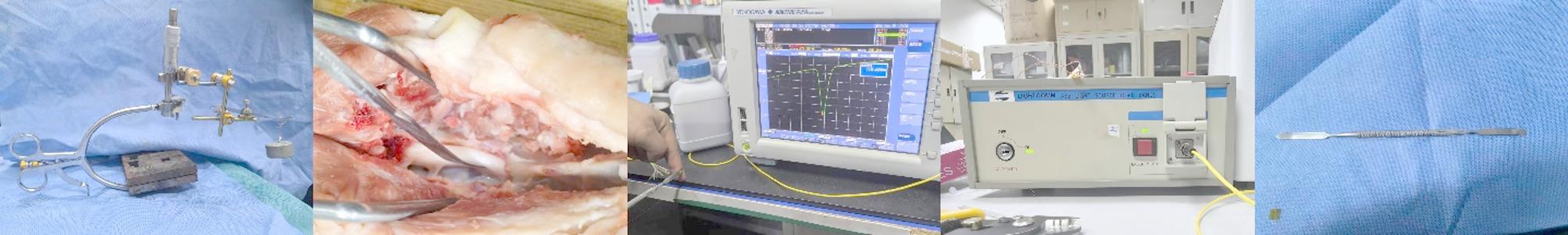
Pre-clinical Validation: in vitro spectrometer calibration and cadaveric testing

**Fig. 5:**
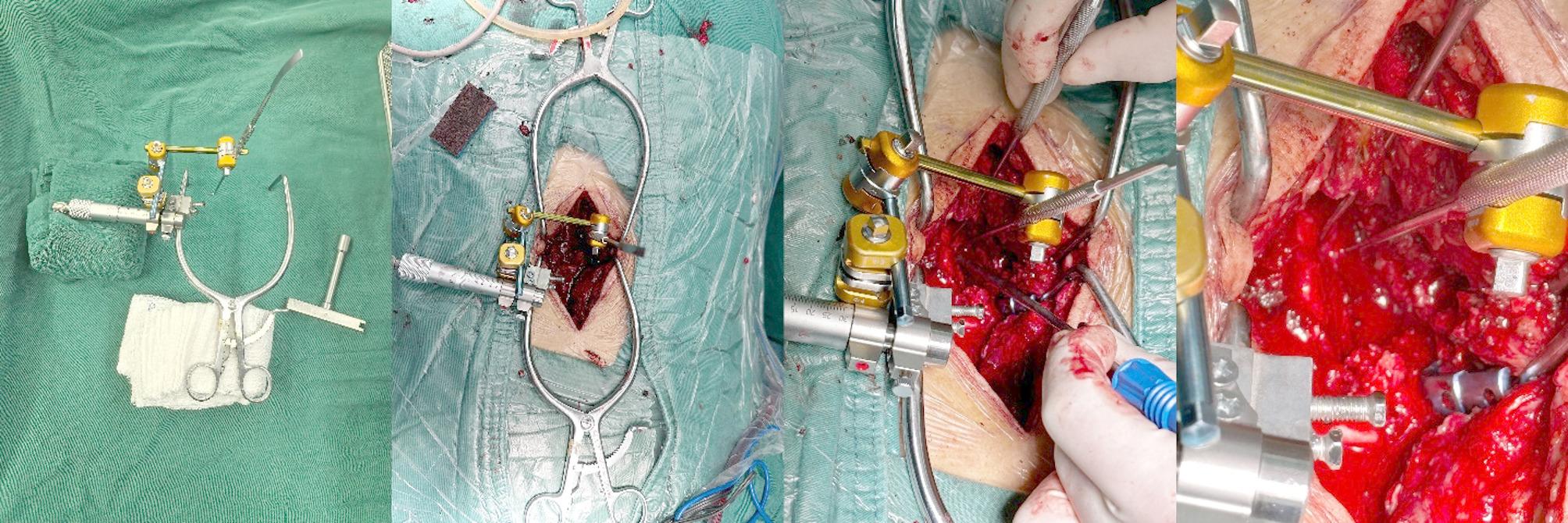
Intraoperative application of the FLDM-NRR system

*FLDM-NRR group*: The FLDM-NRR was used with the associated technical specifications: (1) The device based on the inherent ratchet constant-force mechanism of a micrometer; Following assembly, a force of 3.5 N (± 0.5 N) was measured using external spectrometry and weight testing; (2) Displacement measuring precision: ±0.1 mm; (3) The nerve root displacement distance was determined by the numerical difference value between initial and final retraction positions of the nerve root as displayed on the device; (4) Preoperative calibration of the equipment was conducted to guarantee measurement precision. Subsequently, lateral bone decompression was performed once the nerve root retraction threshold was reached, so as to expand the surgical field (Figs. 3, 4 and 5).

#### Conventional group

Standard nerve root retractors were used, with the force and distance of retraction reliant on the surgeon’s clinical expertise and tactile input.

### Intraoperative neurophysiological monitoring

The FLDM-NRR group received multimodal IONM using the Nicolet Endeavor CR system in accordance with the recommendations of the American Society of Neurophysiological Monitoring (ASNM). Criteria for alerts included: (1) A drop of ≥ 50% in the amplitude of somatosensory evoked potentials (SSEPs) or a lengthening of ≥ 10% in latency; (2) A loss of the amplitude of motor evoked potentials (MEPs) or a sustained decline of ≥ 80%in MEP amplitude; (3) Burst activity in Electromyography (EMG) of ≥ 5 bursts per second or the presence of neurotronic potentials.

**Fig. 6:**
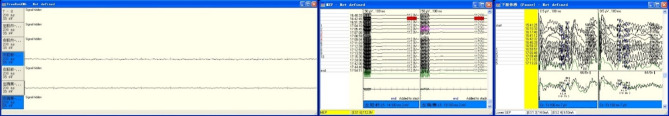
Intraoperative neurophysiological monitoring

Upon the activation of alarms, nerve root retraction was promptly halted, and the surgical was adjusted only once neurophysiological signals had returned to baseline values. All monitoring data were systematically documented and analyzed by experienced neurophysiological technicians in order to ensure inter-operator consistency (Fig. 6).

### Outcome assessment

The primary Outcomes were the frequency of new-onset L5 radiculitis during the three months following surgery and the rate of persisting neurological impairment at the one-year follow-up assessment. L5 radiculitis is characterized by any of the following findings relative to the preoperative baseline: a decrease in muscle strength of ≥ 1 grade in L5-innervated muscle groups (the Medical Research Council [MRC] scale), the emergence of sensory loss or reduced sensation in the L5 dermatome, or the onset or exacerbation of pain in the anatomical distribution of the L5 nerve root. Neurological deterioration was characterized by any newly developed or advancing motor dysfunction, sensory decline, a JOA score reduction of ≥ 3 points from baseline, or the emergence or exacerbation of neurogenic claudication relative to the preoperative state.

The Secondary Outcomes included functional assessment metrics, specifically the Japanese Orthopedic Association (JOA) score (0–29 points), Oswestry Disability Index (ODI, 0–100%), and Visual Analog Scale (VAS) scores for leg and back discomfort (0–10 points). Perioperative parameters included (total operative duration, interbody fusion procedure duration, intraoperative blood loss volume, postoperative drainage volume, duration of hospital stay, and postoperative disc height), device performance metrics included (nerve root retraction distance and rate of necessity for lateral bone decompression), and IONM efficacy parameters included (incidence rate of alerts, signal recovery status, and sensitivity and specificity for predicting postoperative complications).

### Statistical analysis

Continuous variables were presented as mean ± standard deviation (SD) or median (interquartile range [IQR]) and analyzed using Student’s t-test or the Mann-Whitney U test. Categorical variables were summarized as n (%and analyzed using the chi-square (χ²) test or Fisher’s exact test. Effect sizes were reported as relative risk (RR), or mean difference (MD), with 95% confidence intervals (CL). Sensitivity analyses included full case analysis, multivariable logistic regression with covariate adjustment (to account for confounding variables), surgeon-stratified fixed-effects models, monthly calendar coding, and secular-trend control (i.e., calendar quarter + surgeon random intercept). The primary endpoint (3-month L5 radiculitis incidence) was analyzed using multivariable logistic regression under hierarchical testing (α = 0.05); secondary time/functional endpoints were interpreted only if the primary endpoint was statistically significant, with time-trend interaction assessed using the Cochran–Armitage test. Longitudinal trajectories of ODI, JOA, and VAS scores were evaluated using a mixed-effects model to assess the interaction between time and group (time × group). Analyses were performed using SPSS 26.0 (IBM Corp., Armonk, NY, USA) and R 4.5.1, including adjustment for confounding variables, stratified analysis by operating surgeon, and application of two-tailed tests; statistical significance was set at *P* < 0.05, unless otherwise specified.

## Results

### Baseline characteristics

During the study period, 234 patients were enrolled comprising 96 patients in the FLDM-NRR with IONM group and 138 patients in the conventional group. Patient allocation was based on the objective availability of the FLDM-NRR device following its institutional introduction in January 2023, this availability was determined by sterilization cycles and maintenance schedules with no subjective selection criteria applied.

Baseline characteristics demonstrated excellent comparability between the two groups (Table 1). No significant differences were observed in demographics, clinical presentation, or preoperative functional assessment (all *P* > 0.05).

**Table 1:** Baseline demographic and clinical characteristics

Characteristic	FLDM-NRR group (*n* = 96)	Conventional group (*n* = 138)	*P* value
*Demographics*			
Age, years	59.87 ± 7.23	61.15 ± 8.34	0.243
Male sex, n (%)	50 (52.08)	71 (51.45)	0.928
BMI, kg/m²	26.93 ± 2.84	27.38 ± 3.12	0.314
*Clinical presentation*			
Symptom duration, months	27.64 ± 15.27	30.83 ± 16.45	0.182
Neurogenic claudication, n (%)	78 (81.25)	115 (83.33)	0.694
Radicular pain, n (%)	72 (75.00)	108 (78.26)	0.584
*Comorbidities*			
Type 2 diabetes, n (%)	16 (16.67)	27 (19.57)	0.546
Hypertension, n (%)	34 (35.42)	52 (37.68)	0.713
Current/former smoker, n (%)	15 (15.63)	25 (18.12)	0.638
*Functional assessments*			
JOA score (0–29)	11.52 ± 2.83	11.18 ± 2.91	0.407
ODI score (%)	57.84 ± 8.37	59.86 ± 8.94	0.273
VAS back pain score	6.98 ± 1.24	7.42 ± 1.31	0.152
VAS leg pain score	7.73 ± 1.29	7.51 ± 1.38	0.379
*Radiological features*			
Dural sac area, mm²*	80.15 ± 12.53	82.29 ± 13.84	0.327
Preoperative disc height, mm†	6.18 ± 0.87	6.13 ± 0.82	0.689
Severe stenosis (≤ 75 mm²), n (%)	51 (53.13)	64 (46.38)	0.297
Grade III-IV facet degeneration‡, n (%)	72 (75.00)	108 (78.26)	0.563

Data presented as mean ± SD or n (%)

*Measured on axial T2-weighted MRI at most stenotic level

†Measured on preoperative lateral radiographs as the average of anterior, middle, and posterior disc heights at L4-L5 level

‡Graded according to Weishaupt classification for facet joint degeneration

*P* values calculated using independent t-tests for continuous variables and χ² test for categorical variables

### Primary outcomes

In the unadjusted analysis, the FLDM-NRR group significantly reduced L5 radiculitis incidence up to three months after surgery (7/96 [7.29%] vs 25/138 [18.12%], RR 0.40, 95% CI 0.18–0.91, *P* = 0.028; absolute risk reduction 10.83%, number needed to treat 9, 95% CI 2.6–19.1% / 5.2–39.1%) (Table 2).

Time-stratified analysis showed the most profound effect in the first week after surgery 3.13% vs 13.04%, RR 0.24, *P* = 0.020, which gradually waned during the subsequent periods. Cochran–Armitage trend-interaction test revealed a significantly steeper decline of L5 radiculitis over time in the FLDM-NRR group (*P* = 0.032, Table 2). The benefits were not evident at a one-year follow-up, with no corresponding absolute benefit for persistent neurological deficit (ARR 4.1%, 95% CI − 2.0% to 10.2%, *P* = 0.200; RR 0.43, 95% CI 0.12–1.56).

After adjustment for all pre-specified covariates, the protective effect of FLDM-NRR remained significant (adjusted OR 0.31, 95% CI 0.12–0.78, *P* = 0.017; Supplementary_Tables_Sm1). Robustness was confirmed across five sensitivity models (RR 0.24–0.36, all *P* ≤ 0.022; Sy), including surgeon-fixed-effect (or 0.30, *P* = 0.015, Sm2a/2b) and monthly calendar coding (RR 0.24, *P* = 0.006), with no inter-surgeon heterogeneity (χ²=0.02, *P* = 0.99, Sm2b).

**Table 2:** Primary outcomes at 3-month and 1-year follow-up

Primary endpoint	FLDM-NRR Group (*n* = 96)	Conventional group (*n* = 138)	Relative risk (95% CI)	*P* value
*New-onset L5 Radiculitis at 3 Months*				
Overall incidence	7/96 (7.29)	25/138 (18.12)	0.40 (0.18– 0.91)	**0.028**
*Adjusted OR (95% CI)*	–	–	0.31 (0.12–0.78)	**0.017**
*Severity stratification*				
Grade I (Mild)	5/96 (5.21)	16/138 (11.59)	0.45 (0.17–1.20)	0.108
Grade II (Moderate)	2/96 (2.08)	7/138 (5.07)	0.41 (0.09–1.92)	0.259
Grade III (Severe)	0/96 (0.00)	2/138 (1.45)	–	0.535
*Trend-interaction test*	–	–	–	**0.032**
*Symptom categories*				
Pain aggravation (VAS ≥ 2↑) *	4/96 (4.17)	14/138 (10.14)	0.41 (0.14–1.23)	0.112
Sensory deterioration	2/96 (2.08)	8/138 (5.80)	0.36 (0.08–1.66)	0.189
Motor function decline (≥ 1 MRC↓) †	1/96 (1.04)	5/138 (3.62)	0.29 (0.03–2.44)	0.252
*Time-stratified analysis*				
≤ 1 week postoperative	3/96 (3.13)	18/138 (13.04)	0.24 (0.07–0.80)	**0.020**
1 week–1 month	3/96 (3.13)	5/138 (3.62)	0.86 (0.21–3.53)	0.836
1–3 months	1/96 (1.04)	2/138 (1.45)	0.72 (0.07–7.79)	0.786
*Recovery Pattern at 3 Months*				
Complete recovery	6/7 (85.71)	16/25 (64.00)	1.34 (0.81–2.21)	0.252
Partial recovery	1/7 (14.29)	7/25 (28.00)	0.51 (0.07–3.60)	0.493
No improvement	0/7 (0.00)	2/25 (8.00)	–	1.000
*Persistent neurological deterioration at 1 year*				
Any persistent deterioration	3/96 (3.13)	10/138 (7.25)	0.43 (0.12–1.56)	0.200
Persistent motor weakness	1/96 (1.04)	4/138 (2.90)	0.36 (0.04–3.18)	0.361
Persistent sensory deficit	1/96 (1.04)	4/138 (2.90)	0.36 (0.04–3.18)	0.361
Functional deterioration (JOA decline ≥ 3) ‡	0/96 (0.00)	2/138 (1.45)	–	0.535
Persistent/worsened claudication	1/96 (1.04)	2/138 (1.45)	0.72 (0.07–7.79)	0.786
*Long-term recovery analysis*				
Complete neurological recovery at 1 year	93/96 (96.88)	128/138 (92.75)	1.04 (0.99–1.10)	0.111
Residual symptoms affecting daily life	2/96 (2.08)	8/138 (5.80)	0.36 (0.08–1.66)	0.189

*Recovery pattern criteria at 3 months:*

Complete recovery: Complete resolution of L5 radiculitis symptoms, return to baseline neurological function

Partial recovery: ≥50% improvement in symptom severity but residual symptoms persist

No improvement: <50% improvement in symptoms or symptom progression

*Grade for new-onset l5 radiculitis:*

Grade I (Mild): Single symptom, minimal functional impact

Grade II (Moderate): Multiple symptoms or moderate functional limitation

Grade III (Severe): Significant motor deficit with major functional impairment

Data presented as events/total available patients (%). *VAS increase ≥ 2 points from baseline. †Medical Research Council muscle strength scale. ‡Japanese Orthopedic Association score decline. *P* < 0.05 (two-tailed tests). Fisher’s exact test for sparse data. Bold *P* values denote *P* < 0.05 (statsitically significant)

Adjusted OR derived from multivariable logistic regression including age, sex, BMI, baseline pain/ODI/JOA, diabetes, smoking, Lee grade, dural sac area, surgeon (random intercept)

Secondary time-stratified comparisons interpreted under hierarchical testing; trend-interaction test represents the overall time group interaction

### Secondary outcomes

Functional evaluations demonstrated progressive improvements that favored the FLDM-NRR group (Table 3). Early benefits were evident in VAS leg pain scores at one week (4.83 ± 1.94 vs 5.68 ± 2.12 points, *P* < 0.001). At the one-year follow-up, the FLDM-NRR group showed superior ODI scores (18.43 ± 7.85% vs 21.17 ± 8.42%, *P* = 0.018) and sustained reduction in leg and back pain.

MCID analysis showed the FLDM-NRR group had a significantly higher proportion of patients reaching the MCID for VAS-leg pain (95.8% vs 93.5%; *P* = 0.028) and a non-significant trend toward a higher proportion for ODI (87.5% vs 78.3%; *P* = 0.070).

Mixed-effects models revealed significant group effects favoring the FLDM-NRR group for VAS-leg pain (*P* < 0.001) and ODI (*P* < 0.001) across all time points; with larger between-group differences observed at 1 week postoperatively. JOA scores were consistently higher in the FLDM-NRR group (group effect: *P* < 0.001), but there was a minimal time×group interaction (*P* > 0.05) (Supplementary_Tables_Sn1–3).

**Table 3:** Functional outcomes at baseline and follow-up visits

Assessment	FLDM-NRR Group (*n* = 96)	Conventional Group (*n* = 138)	Mean Difference (95% CI)	*P* value
*JOA Score (0–29 points)*				
Baseline	11.52 ± 2.83	11.18 ± 2.91	0.34 (− 0.43 to 1.11)	0.410
1 week postoperative	14.23 ± 3.14	13.87 ± 3.32	0.36 (− 0.58 to 1.30)	0.520
3 months postoperative	21.14 ± 3.67	20.58 ± 4.12	0.56 (− 0.47 to 1.59)	0.340
1 year postoperative	24.87 ± 3.24	24.03 ± 3.65	0.84 (− 0.09 to 1.77)	0.078
*ODI Score (%)*				
Baseline	57.84 ± 8.37	59.86 ± 8.94	− 2.02 (− 5.31 to 1.27)	0.270
1 week postoperative	49.18 ± 9.12	50.79 ± 9.64	− 1.61 (− 4.23 to 1.01)	0.230
3 months postoperative	27.76 ± 8.93	29.42 ± 9.87	− 1.66 (− 4.18 to 0.86)	0.210
1 year postoperative	18.43 ± 7.85	21.17 ± 8.42	− 2.74 (− 5.06 to − 0.42)	**0.018**
VAS Leg Pain Score* (0–10)				
Baseline	7.73 ± 1.29	7.51 ± 1.38	0.22 (− 0.27 to 0.71)	0.380
1 week postoperative	4.83 ± 1.94	5.68 ± 2.12	− 0.85 (− 1.37 to − 0.33)	**< 0.001**
1 month postoperative	3.17 ± 1.73	4.08 ± 1.89	− 0.91 (− 1.38 to − 0.44)	**< 0.001**
3 months postoperative	2.14 ± 1.42	2.57 ± 1.63	− 0.43 (− 0.87 to 0.01)	**0.025**
1 year postoperative	1.18 ± 1.04	1.53 ± 1.17	− 0.35 (− 0.64 to − 0.06)	0.067
*VAS Back Pain Score† (0–10)*				
Baseline	6.98 ± 1.24	7.42 ± 1.31	− 0.44 (− 0.82 to − 0.06)	0.150
1 week postoperative	4.87 ± 1.63	5.16 ± 1.82	− 0.29 (− 0.74 to 0.16)	0.140
3 months postoperative	2.23 ± 1.34	2.58 ± 1.52	− 0.35 (− 0.73 to 0.03)	0.057
1 year postoperative	1.27 ± 0.92	1.59 ± 1.08	− 0.32 (− 0.57 to − 0.07)	0.081
ODI score (MCID > 12%)	84/96 (87.50%)	108/138 (78.26%)		0.070
VAS leg score (MCID > 2)	92/96 (95.83%)	119/138 (93.48%)		**0.028**
VAS Back score (MCID > 2	85/96 (88.54%)	115/138 (83.33%)		0.356

Data presented as mean ± SD. MD, mean difference; CI, confidence interval

JOA, Japanese Orthopedic Association (range 0–29, higher scores indicate better function); ODI, Oswestry Disability Index (range 0–100%, lower scores indicate better function)

*Leg pain including radicular pain and neurogenic claudication symptoms. †Low back pain intensity

*P* values for continuous variables were calculated using independent t-tests; for MCID, using Chi-square tests. Bold *P* values denote *P* < 0.05 (statistically significant)

Secondary endpoints (JOA/ODI/VAS), interpreted via hierarchical testing (primary endpoint *P* = 0.017), no separate *P*-adjustment

### Device performance and IONM efficacy

The FLDM-NRR system maintained 100% adherence to preset force limits (≤ 3.50 N) with zero mechanical failures across 96 procedures (Table 4). The mean nerve root retraction distance was 5.84 ± 1.17 mm. Lateral bone decompression was required in 19.79% of cases due to conditions where the force-limited was reached.

**Table 4:** FLDM-NRR device performance and surgical outcomes (*n* = 96)

Parameter/Outcome	Value	95% CI
*Device performance*		
Force limitation threshold, N	3.50	–
Force threshold violations, n (%)	0 (0.00)	(0.00–3.79)
Mean nerve root displacement, mm	5.84 ± 1.17	(5.60–6.08)
*Surgical adaptations*		
Lateral bone decompression required*, n (%)	19 (19.79)	(12.25–29.22)
Papaverine irrigation applied†, n (%)	3 (3.13)	(0.64–8.83)
Total intraoperative adjustments	18	–
*Safety outcomes*		
Device-related adverse events, n (%)	0 (0.00)	(0.00–3.79)

Data presented as n (%), mean ± SD with 95% CI, or absolute count

*When force limit insufficient for adequate surgical exposure

†Applied for nerve root tension signs or vasospasm prevention

IONM alerts occurred in 9.38% of cases, with signal recovery achieved in 77.78% of these alert cases within 30 min (Table 5). IONM demonstrated high diagnostic accuracy for predicting postoperative complications, with a sensitivity of 85.71% and a specificity of 96.63%,

**Table 5:** Intraoperative neurophysiological monitoring alerts and diagnostic performance (*n* = 96)

Monitoring parameter	Cases (*n*)	Rate (%)	95% CI
*Alert events*			
Any IONM alert*	9	9.38	(4.33–17.12)
SSEP abnormalities†	3	3.13	(0.64–8.83)
MEP abnormalities‡	4	4.17	(1.14–10.31)
Free-running EMG alerts§	5	5.21	(1.70–11.73)
Multiple alert types	2	2.08	(0.25–7.33)
*Alert recovery*			
Complete signal recovery	7	77.78	(40.02–97.19)
Partial signal recovery	2	22.22	(2.81–59.98)
Persistent abnormalities	0	0.00	(0.00–33.63)
*Diagnostic accuracy*			
Sensitivity	–	85.71	(42.13–99.64)
Specificity	–	96.63	(90.36–99.30)
Positive predictive value	–	66.67	(29.93–92.51)
Negative predictive value	–	97.70	(91.92–99.72)

Data presented as n (%) with exact 95% confidence intervals calculated using Clopper-Pearson method

*Any SSEP, MEP, or EMG change meeting predetermined ASNM criteria

†≥50% amplitude decrease or ≥ 10% latency prolongation in tibial/peroneal SSEP

‡≥80% amplitude loss or complete signal loss in lower extremity MEPs

§Burst activity (≥ 5/second) or neurotronic discharges

Signal return to ≥ 90% baseline within 30 min. Recovery times: complete 6.8 ± 3.2 min; partial 15.2 ± 4.1 min

Diagnostic accuracy calculated against postoperative L5 radiculitis as reference standard

### Perioperative safety

No significant differences were observed between the two groups in operative time (132.48 ± 18.73 vs 129.85 ± 21.27 min, *P* = 0.462), intraoperative blood loss (235.64 ± 58.31 vs 248.69 ± 62.47 mL, *P* = 0.241), or duration of hospital stay (11.82 ± 1.19 vs 12.27 ± 1.34 days, *P* = 0.292), thus confirming that the FLDM-NRR technology did not compromise surgical efficiency (Table 6).

**Table 6:** Surgical parameters and perioperative outcomes

Parameter	FLDM-NRR group (*n* = 96)	Conventional group (*n* = 138)	Effect size (95% CI)	*P* value
Operative time (min)	132.48 ± 18.73	129.85 ± 21.27	MD 2.63 (− 4.41 to 9.67)	0.462
Interbody fusion time (min)*	11.24 ± 1.82	10.79 ± 2.08	MD 0.45 (− 0.53 to 1.43)	0.316
Blood loss (mL)	235.64 ± 58.31	248.69 ± 62.47	MD -13.05 (− 34.82 to 8.72)	0.241
Postoperative drainage (mL)	309.87 ± 45.23	315.14 ± 48.62	MD -5.27 (− 16.91 to 6.37)	0.398
Length of stay (days)	11.82 ± 1.19	12.27 ± 1.34	MD -0.45 (− 1.18 to 0.28)	0.292
Postoperative disc height (mm)†	8.93 ± 0.79	8.81 ± 0.72	MD 0.12 (− 0.31 to 0.55)	0.621

Data presented as mean ± SD. MD, mean difference

*Duration from annulus fibrosus incision to cage placement

†Measured on lateral radiographs at 1-week follow-up

P values calculated using independent t-tests

## Discussion

This retrospective study indicates that the FLDM-NRR system combined with IONM significantly reduces the incidence of L5 radiculitis after single-level PLIF from 18% to 7% (absolute risk reduction [ARR]: 10.8%; number needed to treat [NNT]: 9; 95% confidence interval [CI]: 5–39). The neuroprotective effect was particularly significant in the first postoperative week (Cochran–Armitage trend interaction: *P* = 0.032), with this benefit remaining consistent after multivariable adjustment for calendar time, surgeon variability, and all pre-specified covariates—indicating that early mechanical nerve root trauma is the critical trigger for subsequent neuroinflammatory cascades. In our clinical experience, this approach has demonstrated significant utility in addressing one of the most technically challenging aspects of PLIF surgery—striking an optimal balance between adequate neural decompression and the prevention of iatrogenic injury—by providing a quantitative solution.

The identified neuroprotective effects rely on two core principles: meticulous mechanical force regulation and the disruption of pathogenic neuroinflammatory cascades. Biomechanical research indicates that the acceptable limits for lumbar nerve root traction damage consist of individual traction forces below 4.1 ± 0.45 N and cumulative traction loads (force × time) under 42.89 ± 2.96 N·min [16]. Experimental validation verifies that traction forces in the 1–6 N range are within acceptable tolerance limits, with adjacent ligamentous structures providing good protective support [21]. Prior technical validation using fiber-optic sensors in cadaveric studies determined the necessary traction force range of 3.0–4.5 N for secure cage placement. This research determined the 3.5 N threshold to optimize neuroprotection while maintaining surgical practicality, based on both established biomechanical concepts and our clinical surgical expertise. The pathophysiological analysis of the present study indicates that the timing of neuroprotective effects underscores the paramount significance of early prevention of mechanical injury: the most substantial risk reduction occurred within the initial postoperative week (76%), subsequently declining over time, thereby suggesting that averting initial mechanical trauma effectively disrupts neuroinflammatory cascades, including microglial activation and the release of pro-inflammatory cytokine release [22–25].

The FLDM-NRR technology signifies a transition from subjective surgical experience to objective mechanical measurement, in contrast to traditional nerve root retraction approaches. Conventional approaches rely mostly on the surgeon’s clinical expertise and tactile feedback, lacking standardized criteria and thereby leading to considerable inter-operator variability and procedural unpredictability. The FLDM-NRR technique establishes consistent, measurable neuroprotective criteria via real-time mechanical force monitoring and displacement feedback mechanisms.

The relatively elevated incidence of radiculitis in our control group (18.1%) reflects the adoption of more rigorous diagnostic criteria, which include nuanced sensory alterations, pain characteristics, and variations in pain distribution patterns, in contrast to earlier studies that focused primarily on overt motor deficits. Using these same diagnostic criteria, FLDM-NRR group achieved a radiculitis rate of 7.3%, which is lower than the 7.8% nerve root injury rate reported by Okuda et al. [26] and pooled complication rates (>) 5.2% in recent systematic reviews [27, 28], thereby highlighting distinct technological advantages.

The effectiveness study of IONM demonstrated that our integrated monitoring approach achieved 85.7% sensitivity and 96.6% specificity. In contrast to previous systematic evaluations indicating a multimodal monitoring sensitivity of 83.5% and specificity of 93.8% [29–31]. Our analysis indicated slightly enhanced sensitivity and improved specificity, implying benefits in minimizing false-positive warnings and preventing unwarranted surgical procedures. The one-year rates of persistent neurological impairment were 3.1% in the FLDM-NRR group compared to 7.2% in the conventional group (*P* = 0.200). Despite the lack of statistical significance, the 57% relative risk (RR) reduction has significant clinical implications. From a pathophysiological standpoint, enduring neurological impairment is intricately linked to prolonged microglial activation and chronic neuroinflammation [32–35]. Mechanical nerve damage induces microglial activation from a resting state to pro-inflammatory phenotypes, resulting in the release of pro-inflammatory cytokines such as TNF-α and IL-1β, thereby establishing a neuroinflammatory feedback loop [23]. Subsequent to peripheral nerve damage, spinal microglial cells exhibit considerable proliferation and phenotypic alterations in both the dorsal and ventral horn regions, participating in intricate interactions with diverse spinal cellular constituents [34]. The significant decrease in early postoperative radiculitis seen in this study clearly demonstrates the efficacy of FLDM-NRR technology in disrupting pathogenic cascades via the management of initial mechanical damage. In comparison to the current PLIF research, the persistent neurological deterioration rate of our conventional group (7.2%) is within acceptable limits, since literature indicates PLIF neurological damage rates of 9–16% [36]. Conversely, complication rates in our FLDM-NRR cohort were consistently lower than those reported in typical PLIF studies.

### Study limitations

This study is limited by its retrospective, single-Centre design and device allocation based on sterilization cycles rather than randomization; however, concurrent enrolment, calendar-quarter adjustment, and surgeon-stratified sensitivity analyses yielded consistent effect estimates. Early cases during device introduction (January 2023) may reflect surgeon learning curves [37], but calendar-time modelling mitigates this bias. The 10.7% loss-to-follow-up rate and best-/worst-case bounds (RR 0.39–0.50) confirm robustness. The micrometer-based retractor has a fixed 3.5 N threshold (± 0.5 N bench deviation) and measures transmitted rather than tissue-contact force; adjustable or sensor-integrated retractor designs are under development. Multicenter, prospective, randomized trials with ≥ 5-year follow-up are needed to confirm long-term neurological durability and to benchmark FLDM-NRR against other standardized retraction technologies.

### Future directions

Next, multicenter, prospective, randomized trials with ≥ 5-year neurological follow-up should benchmark FLDM-NRR against other standardized retraction technologies. Ultimately, integrating contact-pressure sensors and AI-driven, patient-specific force thresholds will convert the current transmitted-force system into genuine tissue-feedback instrumentation, enabling robotic-platform deployment and segment-specific optimization.

## Conclusions

FLDM-NRR combined with IONM quantitatively reduces the incidence of early L5 radiculitis after PLIF (ARR: 10.8%, NNT: 9, 95% CI: 2.6–19.1%); notably, the proportion of patients achieving the MCID was superior in the FLDM-NRR group for VAS-leg pain scores. However, this approach confers no absolute benefit on persistent neurological deficit at the 1-year follow-up. Next, multicenter, prospective, randomized trials with ≥ 5-year neurological follow-up should benchmark this force-limited strategy against other standardized retraction technologies. Ultimately, integrating contact-pressure sensors and AI-driven, patient-specific force thresholds will convert transmitted-force instrumentation into genuine tissue-feedback systems—enabling robotic-platform deployment and segment-specific optimization for spinal surgery.

## Supplementary Information

Below is the link to the electronic supplementary material.

Supplementary Material 1

Supplementary Material 2

## Data Availability

No datasets were generated or analysed during the current study.
