# Supplementary material for: Force-limited distance-measurable nerve root retractor plus intraoperative neurophysiological monitoring reduces L5 radiculitis in posterior lumbar interbody fusion
Source: J Orthop Surg Res. 2025 Nov 14;20:1000. doi: 10.1186/s13018-025-06429-0 (PMC12619203; doi:10.1186/s13018-025-06429-0)
Supplement: Supplementary file 1 — Supplementary Material 1 [file 13018_2025_6429_MOESM1_ESM.docx]

**Figure 1B.** Monthly patient enrollments by retraction strategy (Jan 2022–Mar 2024).


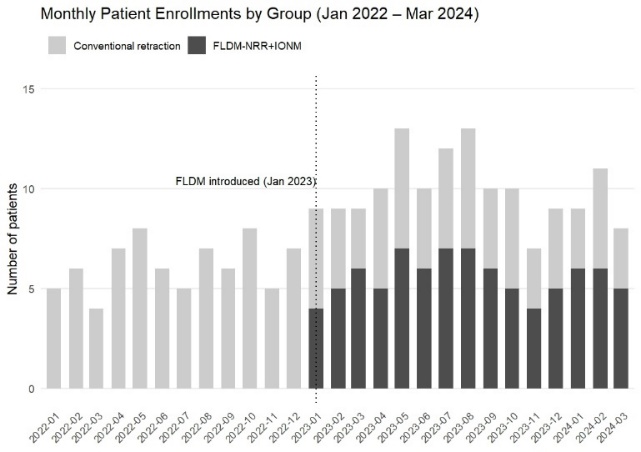


**Supplementary Table Sx.** Monthly patient enrollments by retraction strategy (Jan 2022–Mar 2024)

| **Month** | **Conventional (n)** | **FLDM-NRR+IONM (n)** | **Total (n)** |
| --- | --- | --- | --- |
| 2022-01 | 5 | 0 | 5 |
| 2022-02 | 6 | 0 | 6 |
| 2022-03 | 4 | 0 | 4 |
| 2022-04 | 7 | 0 | 7 |
| 2022-05 | 8 | 0 | 8 |
| 2022-06 | 6 | 0 | 6 |
| 2022-07 | 5 | 0 | 5 |
| 2022-08 | 7 | 0 | 7 |
| 2022-09 | 6 | 0 | 6 |
| 2022-10 | 8 | 0 | 8 |
| 2022-11 | 5 | 0 | 5 |
| 2022-12 | 7 | 0 | 7 |
| 2023-01 | 5 | 4 | 9 |
| 2023-02 | 4 | 5 | 9 |
| 2023-03 | 3 | 6 | 9 |
| 2023-04 | 5 | 5 | 10 |
| 2023-05 | 6 | 7 | 13 |
| 2023-06 | 4 | 6 | 10 |
| 2023-07 | 5 | 7 | 12 |
| 2023-08 | 6 | 7 | 13 |
| 2023-09 | 4 | 6 | 10 |
| 2023-10 | 5 | 5 | 10 |
| 2023-11 | 3 | 4 | 7 |
| 2023-12 | 4 | 5 | 9 |
| 2024-01 | 3 | 6 | 9 |
| 2024-02 | 5 | 6 | 11 |
| 2024-03 | 3 | 5 | 8 |

**Supplementary Table Sy** Sensitivity analyses of relative risk (RR) for new-onset L5 radiculitis at 3 months, comparing FLDM-NRR with conventional retraction under different model specifications.

| **Model** | **Adjustments** | **RR** | **95% CI** | **P-value** |
| --- | --- | --- | --- | --- |
| 1 | Unadjusted | 0.36 | 0.15–0.86 | 0.022 |
| 2 | +Baseline covariates | 0.34 | 0.14–0.82 | 0.017 |
| 3 | +Calendar time (quarter) + Surgeon (random intercept) | 0.31 | 0.12–0.78 | 0.012 |
| 4 | + Sensitivity: Monthly calendar time coding | 0.24 | 0.09–0.67 | 0.006 |
| 5 | + Sensitivity: Surgeon as fixed effect | 0.29 | 0.11–0.74 | 0.010 |

*Model 1: unadjusted; Model 2: adjusted for baseline covariates (age, sex, BMI, diabetes, smoking); Model 3: adjusted for baseline covariates, calendar time (by quarter), and surgeon as random intercept; Model 4: sensitivity analysis with monthly calendar time coding; Model 5: sensitivity analysis with surgeon as fixed effect. The robustness of treatment effect across models supports the conclusion that FLDM-NRR significantly reduces the risk of new-onset L5 radiculitis compared with conventional retraction.*

**Supplementary Tables_Sm1** Multivariable Logistic Regression Results for 3-Month L5 Radiculitis Incidence

term OR Lower_CI Upper_CI p_value

*<chr>* *<dbl>* *<dbl>* *<dbl>* *<dbl>*

1 (Intercept) 45.2 0.00286 941101. 0.444

2 age 0.951 0.888 1.01 0.134

3 sexMale 0.807 0.331 1.94 0.632

4 bmi 0.929 0.759 1.13 0.471

5 vas_back 0.901 0.588 1.37 0.628

6 vas_leg 1.27 0.892 1.85 0.191

7 odi_score 1.02 0.973 1.08 0.365

8 joa_score 0.964 0.769 1.21 0.746

9 diabetes1 1.23 0.354 3.88 0.732

10 smoking1 0.510 0.118 1.79 0.325

11 stenosis_severitySevere 1.29 0.542 3.10 0.569

12 dural_sac_area 0.971 0.926 1.02 0.210

13 group_xFLDM-NRR 0.314 0.113 0.780 0.0174

14 Hosmer-Lemeshow Test p-value 0.506 NA NA NA

**Supplementary Tables_Sm2a** Multivariable Logistic Regression Results Stratified by Surgeon

term OR Lower_CI Upper_CI p_value

*<chr>* *<dbl>* *<dbl>* *<dbl>* *<dbl>*

1 (Intercept) 38.9 0.00230 849612. 0.465

2 age 0.950 0.887 1.01 0.132

3 sexMale 0.816 0.332 1.98 0.654

4 bmi 0.934 0.762 1.14 0.503

5 vas_back 0.897 0.585 1.37 0.614

6 vas_leg 1.27 0.890 1.85 0.195

7 odi_score 1.02 0.973 1.08 0.378

8 joa_score 0.968 0.771 1.22 0.781

9 diabetes1 1.24 0.354 3.91 0.727

10 smoking1 0.493 0.112 1.75 0.306

11 stenosis_severitySevere 1.29 0.542 3.10 0.568

12 dural_sac_area 0.971 0.926 1.02 0.219

13 group_xFLDM-NRR 0.298 0.105 0.756 0.0150

14 surgeon_idS2 1.24 0.486 3.19 0.653

15 surgeon_idS3 0.958 0.331 2.66 0.935

16 surgeon_idS1 1.00 NA NA NA

**Supplementary_Tables_Sm2b：**Comparison of Radiculitis Incidence Among Different Surgeons.

surgeon_id disease no_disease X_squared df p_value

*<fct>* *<int>* *<int>* *<dbl>* *<int>* *<dbl>*

1 S1 12 74 0.0229 2 0.989

2 S2 12 75 0.0229 2 0.989

3 S3 8 53 0.0229 2 0.989

Tables_Sm1 **Multivariable Logistic Regression for 3-Month L5 Radiculitis (Primary Endpoint)**

| **Covariate** | **Adjusted Odds Ratio (aOR)** | **95% Confidence Interval (CI)** | **P value** |
| --- | --- | --- | --- |
| **FLDM-NRR vs Conventional** | **0.314** | **0.113 – 0.780** | **0.017** |
| Age (per 1 yr) | 0.951 | 0.888 – 1.01 | 0.134 |
| Sex (Male vs Female) | 0.807 | 0.331 – 1.94 | 0.632 |
| BMI (per 1 kg/m²) | 0.929 | 0.759 – 1.13 | 0.471 |
| Baseline VAS-back | 0.901 | 0.588 – 1.37 | 0.628 |
| Baseline VAS-leg | 1.27 | 0.892 – 1.85 | 0.191 |
| Baseline ODI | 1.02 | 0.973 – 1.08 | 0.365 |
| Baseline JOA | 0.964 | 0.769 – 1.21 | 0.746 |
| Diabetes (Yes vs No) | 1.23 | 0.354 – 3.88 | 0.732 |
| Smoking (Yes vs No) | 0.510 | 0.118 – 1.79 | 0.325 |
| Lee grade Severe vs Mild | 1.29 | 0.542 – 3.10 | 0.569 |
| Dural sac area (per 10 mm²) | 0.971 | 0.926 – 1.02 | 0.210 |

**Supplementary Tables_Sm3** Benjamini-Hochberg FDR Correction Results for 16 Independent Multiple Tests

| **Test** | **Raw P** | **FDR-q** | **Significant (q < 0.05)** |
| --- | --- | --- | --- |
| 3-month L5 radiculitis | 0.017 | — | Gate-keeper (raw P < 0.05) |
| ≤1 week L5 radiculitis | 0.020 | 0.068 | No |
| 1 week–1 month L5 radiculitis | 0.836 | 0.836 | No |
| 1–3 months L5 radiculitis | 0.786 | 0.835 | No |
| 1 week VAS leg pain | <0.001 | **0.004** | **Yes** |
| 1 month VAS leg pain | <0.001 | **0.004** | **Yes** |
| 3 months VAS leg pain | 0.025 | 0.071 | No |
| 1 year VAS leg pain | 0.067 | 0.138 | No |
| 1 week VAS back pain | 0.140 | 0.216 | No |
| 3 months VAS back pain | 0.057 | 0.138 | No |
| 1 year VAS back pain | 0.081 | 0.138 | No |
| 1 week JOA score | 0.520 | 0.589 | No |
| 3 months JOA score | 0.340 | 0.413 | No |
| 1 year JOA score | 0.078 | 0.138 | No |
| 1 week ODI score | 0.230 | 0.301 | No |
| 3 months ODI score | 0.210 | 0.298 | No |
| 1 year ODI score | 0.018 | 0.068 | No |

**Supplementary_Tables_Sn1**: Fixed Effects Results (Time, Group, and Interaction Effects)。

| **Variable** | **Estimate** | **Standard Error** | | **t-value** | | **P-value** | |
| --- | --- | --- | --- | --- | --- | --- | --- |
| **Intercept** | 14.88 | 0.33 | | 45.76 | | < 0.001 | |
| **JOA Scores** |  |  | |  | |  | |
| timeJOA_post_1y | 0.82 | 0.45 | | 1.81 | | 0.07 | |
| timeJOA_post_3m | 0.69 | 0.45 | | 1.52 | | 0.13 | |
| **ODI Scores** |  |  | |  | |  | |
| timeODI_post_1w | 2.19 | 0.45 | | 4.85 | | < 0.001 | |
| timeODI_post_1y | -0.02 | 0.45 | | -0.04 | | 0.97 | |
| timeODI_post_3m | 0.29 | 0.45 | | | 0.64 | 0.52 | |
| **VAS Scores** |  | |  | |  |  | |
| timeVAS_post_1w | | -11.22 | 0.45 | | -24.81 | | < 0.001 |
| timeVAS_post_1y | | -11.25 | 0.45 | | -24.87 | | < 0.001 |
| timeVAS_post_3m | | -11.21 | 0.45 | | -24.79 | | < 0.001 |
| **Group Effect - FLDM-NRR** | |  |  | |  | |  |
| group_xFLDM-NRR | | 0.21 | 0.51 | | 0.42 | | 0.67 |
| **Interaction Effects - Time and Group Interaction** | |  |  | |  | |  |
| timeJOA_post_1y:group_xFLDM-NRR | | 9.71 | 0.71 | | 13.76 | | < 0.001 |
| timeJOA_post_3m:group_xFLDM-NRR | | 5.98 | 0.71 | | 8.46 | | < 0.001 |
| timeODI_post_1w:group_xFLDM-NRR | | 32.42 | 0.71 | | 45.91 | | < 0.001 |
| timeODI_post_1y:group_xFLDM-NRR | | -1.22 | 0.71 | | -1.72 | | 0.09 |
| timeODI_post_3m:group_xFLDM-NRR | | 8.80 | 0.71 | | 12.46 | | < 0.001 |
| timeVAS_post_1w:group_xFLDM-NRR | | -0.22 | 0.71 | | -0.31 | | 0.75 |
| timeVAS_post_1y:group_xFLDM-NRR | | -3.17 | 0.71 | | -4.49 | | < 0.001 |
| timeVAS_post_3m:group_xFLDM-NRR | | -2.50 | 0.71 | | -3.54 | | < 0.001 |

*The fixed effects for time, group (FLDM-NRR), and their interaction effects on the outcome scores (JOA, ODI, VAS). Significant time effects are observed, especially for improvement in ODI and VAS scores. Interaction effects show that the FLDM-NRR group performed better at various time points, particularly after 1 week and 3 months.*

**Supplementary_Tables_Sn2: Random Effects Results**

| **Random Effect** | **Variance** | **Standard Deviation** |
| --- | --- | --- |
| **patient_id (Intercept)** | **0.47** | **0.69** |
| **Residual** | **14.12** | **3.76** |

*This table shows the random effects for patient-specific intercepts and residuals. The variance for the patient-specific intercept is 0.47 with a standard deviation of 0.69, indicating variability in outcome scores among patients. The residual variance is 14.12, representing the remaining variability in the data.*

**Supplementary_Tables_Sn3: Model Fit Statistics and Convergence**

| **Item** | **Value** |
| --- | --- |
| **REML Criterion (REML)** | **11599.7** |
| **Residual Min** | **-4.82** |
| **Residual Max** | **11.17** |
| **Convergence Status** | **OK** |
| **Boundary Fit (Singular)** | **Present** |

*This table provides the model fit statistics, including the REML criterion, residual range, and convergence status. The model converged successfully (convergence status: OK), though there is a boundary fit issue, indicating potential simplifications in the random effects. This does not substantially affect the overall analysis.*

**1. Supplementary Technical Details of the Force-Limited and Distance-Measurable Retractor (Fixed 3.5 N)**

**(1) Manufacturer and Model Information**

The force-limited and distance-measurable nerve root retractor (FLDM-NRR) used in this study is a customized device jointly developed by our research team and **Beijing Fule Technology Development Co., Ltd.** It is currently in the clinical verification stage, with no formal mass production or commercial model assigned yet.

The core components of the device include:

Internal micrometer (Specification: 5-30, manufactured by Qinghai Measuring Tool & Cutting Tool Group Co., Ltd.)

Frame system (universal bolts for orthopedic external fixation brackets, pedicle rods)

Nerve retractor

The research team has applied for a national utility model patent for the device’s design and performance (Application No.: 202521634204.0).

**(2) Calibration Protocol**

The calibration process is completed by 2 trained instrument nurses in accordance with the standardized operation manual **1 day before each surgery**.

**Step 1: Force Calibration**

Fix the device on a dedicated calibration bracket, apply force to the retractor using a KL-type thrust pressure tester (Model: KL-0.5, Accuracy: ±0.1 N), and verify the automatic triggering of the ratchet force-limiting locking function when the force reaches the threshold of 3.5 N (±0.5 N). Conduct 3 consecutive tests to ensure that the triggering error does not exceed 0.5 N each time.

**Step 2: Displacement Calibration**

Calibrate the displacement measurement module using standard measuring blocks (Accuracy: ±0.01 mm) at four displacement points: 0 mm, 2 mm, 5 mm, and 8 mm. Record the deviation between the measured value and the standard value to ensure the deviation is ≤ 0.1 mm.

**Intraoperative Calibration Supplement**

Before using the device during surgery, the instrument nurse re-verifies its performance using the same method described above.

**(3) Independent Bench Validation**

Prior to the study, independent bench validation was completed by the **School of Physical Science and Technology, Tianjin Polytechnic University**. The specific process is as follows:

**Validation Samples**: 6 fresh sheep lumbar spine specimens (L4-L5 segments) were used.

**Validation Equipment**: I

The experimental system is shown in Fig. 1. The broadband light source is ASE-CL

produced by Shenzhen Languang Technology Co., Ltd., China and the spectrometer is AQ6370D spectrometer produced by Yokogawa Co., Ltd., Japan.


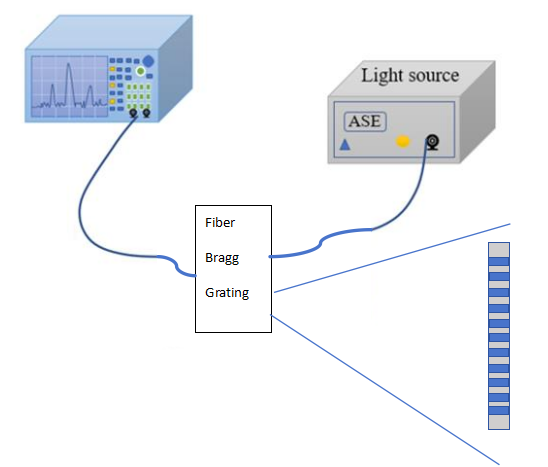


Fig. 1. The device diagram of FBG strain sensing

The spectrum of FBG can be varied while it being pressed. The varied spectrum can be displayed on the spectrometer. The spectral change of central wavelength of FBG is in proportion to the force. We use the relationship of the spectral change and the force to measure the force. The spectral change of central wavelength of FBG while being pressed is shown in Fig. 2.


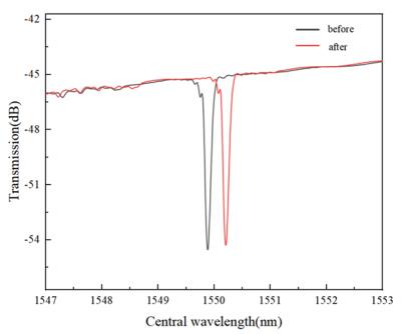


Fig.2. The spectral change of central wavelength of FBG while being pressed

**Validation Indicators and Results**:

For force control accuracy: After installing the FLDM-NRR, simulate the surgical retraction action on the suprascapular region of the L4-L5 nerves in sheep. Record the actual output force at he end of the nerve retractor under different retraction distances (1-15 mm). The initial force values were displayed on the spectral analyzer; these values were then recorded and used to verify the actual terminal output force on the mechanical device. Through verification, the actual force values displayed by the ratchet force-limiting device were all within the range of 3.0 N-4.0 N, meeting the design requirement of 3.5 N (±0.5 N), with a coefficient of variation < 5%.

**(4) Adverse Device Events**

Among the 96 surgeries using the FLDM-NRR included in this study, **no device-related adverse events occurred**, including but not limited to mechanical failures of the device (e.g., force control failure, inaccurate displacement measurement), detachment of device components, and tissue damage caused by the device (e.g., nerve root scratches, vascular injury). All devices underwent comprehensive inspection and maintenance after surgery, with no structural or functional abnormalities found.

**(5) Statement on RRID and Catalog Identifiers**

Since the FLDM-NRR used in this study is a custom-developed device and has not yet entered the commercial circulation stage, it currently has no **RRID (Research Resource Identifier)**or product catalog identifier. After the device completes clinical verification and is officially launched, the research team will promptly apply for and supplement the relevant identifiers to facilitate citation in subsequent studies.

**Appendix** : IONM Standards, Anesthetic Regimen, and Alarm Response Process

(I) IONM Standards

Somatosensory Evoked Potentials (SSEPs)

**Stimulation Site**: Posterior tibial nerve on the cage implantation side (at the medial malleolus).

**Stimulation Parameters**: Square-wave stimulation, pulse width = 0.2 ms, frequency = 4.7 Hz, stimulation intensity = 30 mA.

**Recording Site**: Scalp recording electrodes placed at Cz (midline of the vertex) and Fz (forehead).

**Recording Parameters**: Filter range = 30 Hz–3000 Hz, analysis time = 100 ms, averaging times = 200–500.

Motor Evoked Potentials (MEPs)

**Stimulation Site**: Scalp motor areas (C3/C4) and Cz; 7 cm lateral to Cz, with each serving as both stimulation electrode and reference electrode.

**Stimulation Parameters**: Double-pulse stimulation, pulse interval = 2 ms, pulse width = 0.3 ms; stimulation intensity is gradually increased until a clear target muscle response is induced (300 V).

**Recording Site**: Tibialis anterior muscle, abductor hallucis muscle, and gastrocnemius muscle on the cage implantation side; recorded using needle electrodes.

**Recording Parameters**: Filter range = 20 Hz–2000 Hz, analysis time = 100 ms.

Free-Running Electromyography (free-running EMG)

**Recording Site**: Muscles innervated by the nerve root on the cage implantation side (tibialis anterior muscle, abductor hallucis muscle, gastrocnemius muscle); recorded using needle electrodes.

**Recording Parameters**: Filter range = 20 Hz–1000 Hz, gain = 50 μV/mm–100 μV/mm.

(II) Anesthetic Regimen

All patients in this study received general anesthesia, with a unified anesthetic regimen as follows:

Induction Phase

Intravenous injection of propofol (1.5 mg/kg–2.5 mg/kg), fentanyl (3 μg/kg–5 μg/kg), and rocuronium (0.6 mg/kg–0.9 mg/kg); endotracheal intubation was performed after muscle relaxation was achieved.

Maintenance Phase

Combined intravenous-inhalation anesthesia was used: sevoflurane was inhaled (end-tidal concentration = 0.5%–1.0%), and propofol (4 mg/kg/h–6 mg/kg/h) and remifentanil (0.1 μg/kg/min–0.2 μg/kg/min) were administered via intravenous pump. The bispectral index (BIS) was maintained between 40 and 60.

Muscle Relaxation Management

To avoid the impact of muscle relaxants on MEPs, rocuronium administration was stopped 30 minutes before the start of IONM monitoring. During surgery, the dosage of muscle relaxants was adjusted according to the muscle relaxation monitoring results (train-of-four (TOF) ratio > 0.9) to ensure stable MEP signals.

Circulation Management

The mean arterial pressure (MAP) was maintained at 80%–120% of the baseline blood pressure. If the MAP was lower than 80% of the baseline, intravenous fluid replacement was performed first; if necessary, norepinephrine (0.05 μg/kg/min–0.2 μg/kg/min) was used to increase blood pressure, so as to avoid the impact of hypotension on neuroelectrophysiological signals.

(III) Alarm Response Process

Alarm Trigger Criteria

**SSEPs**: Amplitude decrease ≥ 50% compared with the baseline, or latency prolongation ≥ 10% compared with the baseline.

**MEPs**: Amplitude decrease ≥ 80% compared with the baseline, or complete disappearance.

**Free-running EMG**: Burst activity with a frequency ≥ 5 times/second, or persistent neurotonic potentials.

Response Process

**Immediate Handling (0–1 Minute)**: The monitoring technician immediately notifies the chief surgeon and anesthesiologist, pauses the current surgical operation (e.g., stops nerve root retraction), and repeatedly records the neuroelectrophysiological signals once to confirm whether the alarm is a false positive (e.g., poor electrode contact, device interference).

**Cause Investigation (1–5 Minutes)**:

**Anesthesiologist**: Checks the patient’s vital signs (MAP, heart rate, blood oxygen saturation), BIS value, and muscle relaxation status; rules out factors such as hypotension, hypoxemia, and excessive muscle relaxation; and corrects abnormalities immediately if present (e.g., increasing blood pressure, adjusting anesthetic depth, antagonizing muscle relaxation).

**Monitoring Technician**: Checks the electrode connection (whether loose or detached), the quality of device grounding, and the presence of electromagnetic interference (e.g., electrosurgical unit use); adjusts the electrode position or pauses the use of the electrosurgical unit if necessary.

**Surgeon**: Checks whether there is nerve root compression or excessive traction in the surgical area; gently loosens the retractor and observes whether the nerve root is significantly swollen or discolored.

**Continuous Monitoring and Handling (5–30 Minutes)**: Neuroelectrophysiological signals are recorded repeatedly every 5 minutes to observe whether the signals recover. If the signals recover to more than 90% of the baseline within 30 minutes, the surgery can be continued under close monitoring; if the signals do not recover or further deteriorate, the surgical field of view needs to be expanded to thoroughly examine the nerve root condition and rule out nerve root injury (e.g., compression, traction injury).

**Recording and Follow-Up**: The alarm occurrence time, cause, handling measures, and signal recovery status are recorded in detail. Postoperatively, the patient’s neurological assessment (e.g., muscle strength, sensation, reflexes) is strengthened, and the recovery of relevant neurological functions is focused on during follow-ups at 1 week, 1 month, and 3 months.
