# Supplementary material for: Force-limited distance-measurable nerve root retractor plus intraoperative neurophysiological monitoring reduces L5 radiculitis in posterior lumbar interbody fusion
Source: J Orthop Surg Res. 2025 Nov 14;20:1000. doi: 10.1186/s13018-025-06429-0 (PMC12619203; doi:10.1186/s13018-025-06429-0)
Supplement: Supplementary file 2 — Supplementary Material 2 [file 13018_2025_6429_MOESM2_ESM.docx]

**Force-Limited Distance-Measurable Nerve Root Retractor Plus Intraoperative Neurophysiological Monitoring Reduces** **L5 Radiculitis in Posterior Lumbar Interbody Fusion**

Min Wang^1,2^, Zhiqing Chen^3^, Jibin Ma^4^, Yufei Yuan^5^, Jun Miao^6^*, Wangqiang Wen^7^, Bangquan Liao^8^*

* Corresponding authors: Jun Miao, Email: mj6688@163.com; Bangquan Liao, Email: [liaobangquan@163.com](mailto:liaobangquan@163.com).

1.Clinical School/College of Orthopedics, Tianjin Medical University, No. 22 Qixiangtai Road, Heping District, Tianjin, China.

2.Department of Orthopedics (Spine Division), Beichen Hospital Affiliated to Nankai University, No. 7 Beiyi Road, Beichen District, Tianjin, China.

3.Clinical College of Orthopedics, Tianjin Medical University, 406 Jiefang South Road ,Hexi District,Tianjin,China.

4.Department of Orthopedics, The Second People's Hospital of Changzhi, No. 83 Peace West Street, Luzhou District, Changzhi, Shanxi Province, China.

5.The orthopedic surgery department of HanDan Central Hospital, 15 Zhonghua South Street, Handan 056001,Hebei, China.

6.Department of Spine Surgery, Tianjin Hospital, Tianjin University, Jiefangnanlu 406, Hexi District, Tianjin, China.

7.The First Affiliated Hospital of Hainan Medical University (Hainan Province Clinical Medical Center, National Clinical Key Specialty Construction Department), Haikou, Hainan, China.

8. School of Physical science and technology, Tiangong University, Tianjin, China.

**Abstract**

**Objective:** To evaluate the incidence of L5 radiculitis in patients undergoing posterior lumbar interbody fusion (PLIF) using a force-limited distance-measurable nerve root retractor (FLDM-NRR) combined with intraoperative neurophysiological monitoring (IONM), compared with conventional nerve root retraction.

**Methods:** This retrospective cohort study examined 234 patients with L4-L5 lumbar spinal stenosis (LSS) undergoing single-level PLIF from January 2022 to March 2024. Patients were categorized into the FLDM-NRR plus IONM group (n=96) with force limitation ≤3.5N, and the conventional nerve root retraction group (n=138). The primary outcome was 3-month L5 radiculitis; Secondary outcomes included 1-year persistent neurological impairment, Japanese Orthopedic Association (JOA) scores, Oswestry Disability Index (ODI), Visual Analog Scale (VAS) pain scores, and achievement of minimal clinically important difference (MCID). Multivariable logistic regression, mixed-effects models, and hierarchical testing were applied.

**Results** FLDM-NRR with IONM reduced the 3-month L5 radiculitis incidence (7.29% vs. 18.12%, adjusted OR = 0.31, 95% CI 0.11–0.78; P = 0.017; absolute risk reduction(ARR) = 10.83%; number needed to treat(NNT) = 9), with greatest protection in the first postoperative week (3.13% vs. 13.04%, P=0.020). No significant difference was observed in 1-year persistent neurological impairment (3.13% vs 7.25% P=0.200). The FLDM-NRR group showed superior ODI (18.43±7.85% vs. 21.17±8.42%, P=0.018) and higher MCID rates in VAS leg pain (95.8% vs. 93.5%, P=0.028). IONM demonstrated 85.71% sensitivity and 96.63% specificity in predicting postoperative complications. The FLDM-NRR system consistently upheld the predetermined force threshold in all instances without any device-related problems.

**Conclusion**

FLDM-NRR combined with IONM significantly reduces early L5 radiculitis after PLIF and provides short-term functional benefits. Long-term neuroprotection remains unproven and requires validation in prospective randomized trials.

**Keywords:** Lumbar spinal stenosis; Posterior lumbar interbody fusion; Nerve root protection; Intraoperative neurophysiological monitoring; Force-limited nerve root retractor; L5 Radiculitis

**Introduction**

Lumbar spinal stenosis (LSS) represents a common degenerative spinal condition widespread among the elderly, with clinical incidence rates between 11% and 39% [1]. This syndrome is defined by the constriction of the spinal canal, lateral recess, or neural foramina, leading to neurogenic claudication, radicular discomfort, and increasing functional deterioration [2]. When conservative therapy is ineffective, posterior lumbar interbody fusion (PLIF) serves as the conventional surgical intervention for symptomatic LSS [3,4].

Neurological issues associated with surgery considerably affect patient outcomes in PLIF surgeries. Research indicates that PLIF has a nerve root damage risk of up to 7.8%, significantly above the 2.0% rate linked to transforaminal lumbar interbody fusion (TLIF) [5,6]. *Endoscopic techniques have further been shown to reduce nerve-related complications via precise nerve root protection The L5 nerve root is particularly susceptible to injury during surgical procedures, primarily due to its elongated course beneath the neural foramen and close proximity to adjacent tissues [7, 8].* [7,8(1)](1). Injury may arise from traction, compression, or severe retraction during fusion operations. Traditional retraction methods rely on the surgeon's expertise and tactile perception, lacking objective quantitative benchmarks and resulting in inter-operator variability.

However, Intraoperative neurophysiological monitoring (IONM) offers real-time neurological evaluation using multimodal approaches and is the prevailing standard for neuroprotection in spine surgery [9,10]. IONM demonstrates specific limitations in identifying mechanical traction injuries, since electrophysiological alterations may trail after the actual nerve damage [11]. The time delay, exacerbated by IONM's vulnerability to anesthetic and hemodynamic disruptions, may permit irreparable damage to transpire prior to detection [12]. These constraints have stimulated interest in supplementary mechanical protection techniques.

The force-limited , distance-measurable nerve root retractor (FLDM-NRR) was developed based on biomechanical principles to limit nerve root retraction force to *≤*3.5N while enabling real-time measurement of retraction displacement. Biomechanical research indicates that neural tissues possess defined tolerance levels to mechanical stress, beyond which permanent damage ensues [13-16]. In 2023, our hospital adopted FLDM-NRR technology with IONM as a component of our neuroprotection strategy for PLIF surgeries.

Clinical data concerning the efficacy of force-controlled nerve root protection is limited. Previous studies  *have largely focused* on biomechanical analyses and small case series, and large-scale clinical *evaluations comparing outcomes* between force-limited and traditional retraction *techniques remain insufficient.*. The appropriate combination of mechanical force control with electrophysiological monitoring has not been thoroughly evaluated in clinical practice.

The *implementation* of FLDM-NRR technology at our institution enabled a comparison of results with our prior standard practice using traditional retraction procedures. This retrospective cohort research *evaluates* the clinical efficacy of FLDM-NRR *combined* with IONM, compared *with* conventional retraction, in *reducing the risk of* postoperative L5 radiculitis in patients *undergoing single-level L4-L5 PLIF for LSS.*. *It was hypothesized that*  force-controlled nerve root retraction would reduce *the incidence of* postoperative L5 radiculitis while *maintaining*  surgical *efficacy*. *Ultimately, this study aims to* provide clinical evidence  *supporting the application of standardized mechanical neuroprotective strategies in spinal surgery.*

**Methods**

**Study Design and Population**

This retrospective cohort study analyzed clinical data from patients who underwent single-level L4-L5 PLIF at our spinal surgery department between January 2022 and March 2024. The protocol was approved by our institutional review board (IRB), and all procedures complied with the Declaration of Helsinki and relevant international medical ethical standards. For this study, a total of 234 patients were included who underwent single-level L4-L5 PLIF and met the inclusion criteria throughout the study period.

**Inclusion criteria:** aged 40 to 80 years with single-level L4-L5 moderate to severe LSS, as confirmed by magnetic resonance imaging (MRI). The severity of stenosis was evaluated using the Lee grading criteria¹⁷, with a dural sac cross-sectional area of ≤100 mm². The supplementary inclusion criteria included: (1) symptomatic neurogenic claudication or radicular pain refractory to conservative treatment for ≥3 months; (2) American Society of Anesthesiologists (ASA) classification ≤II; (3) undergoing PLIF surgery; (4) clinical data being accessible during preoperative, postoperative, and follow-up periods.

**Exclusion criteria:** (1) a previous lumbar surgery at the same level; (2) preoperative presence of spinal infection, neoplasm, or acute fracture; (3) significant comorbidities that impair neurological evaluation (severe diabetic peripheral neuropathy, myelopathy, severe peripheral vascular disease); (4) inadequate clinical documentation or missing follow-up information.

Patients were allocated to neuroprotection strategies based on objective FLDM-NRR device availability following its institutional introduction in January 2023. Device accessibility was determined by sterilization cycles and maintenance schedules, with no subjective selection criteria influencing group assignment. Clinical data showed that a total of 234 patients were enrolled: 96 in the FLDM-NRR with IONM group and 138 in the traditional nerve root retraction group. (**Figure1A**) Due to intermittent device availability, both strategies were used concurrently from January 2023 to March 2024, ensuring contemporaneous recruitment. A calendar timeline of monthly enrollments is shown (**Figure 1B)**, with detailed counts provided in **Supplementary Table Sx**.

**Figure 1A.** CONSORT Flow Diagram for Patient Selection and Allocation

[
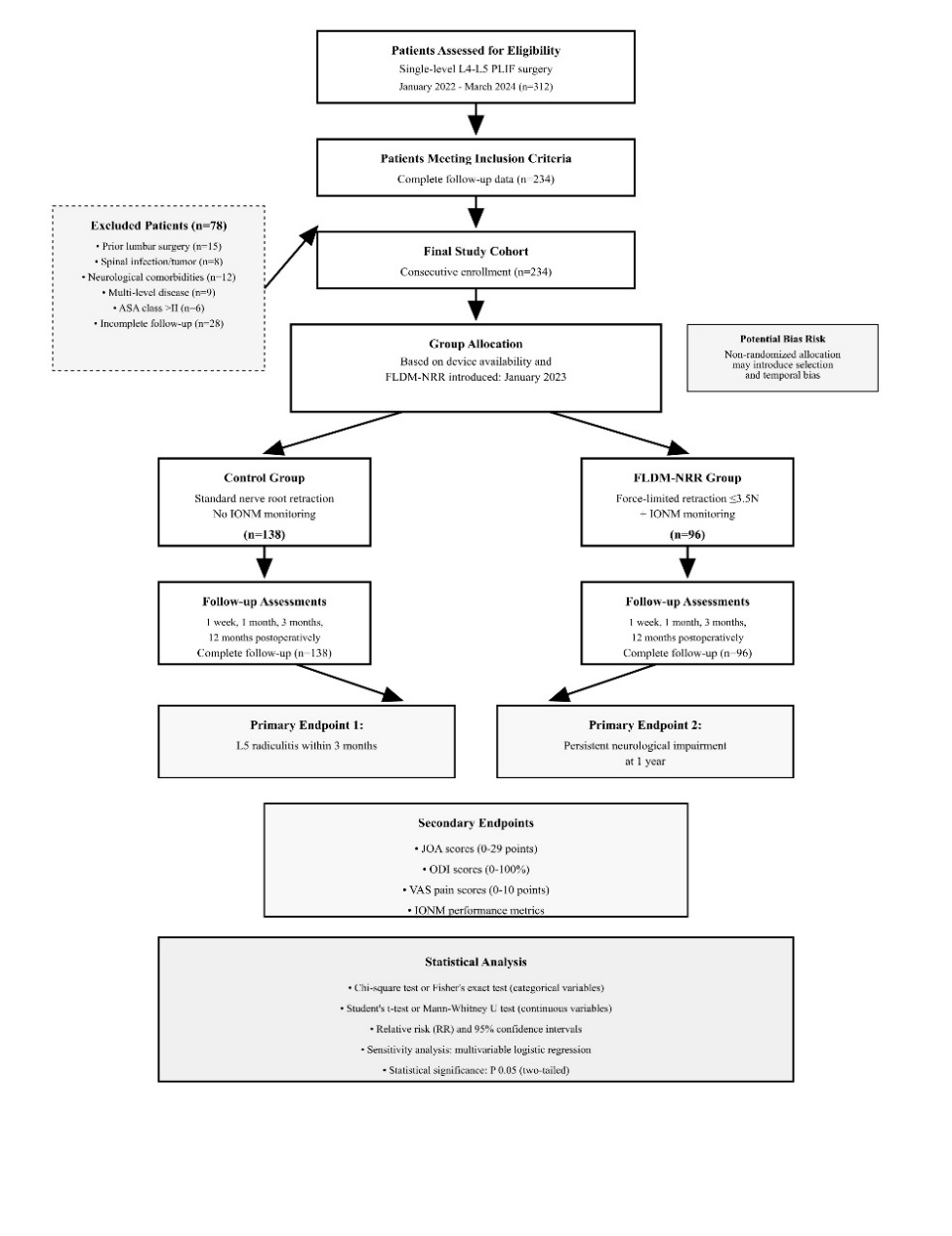
]

**Figure 1B. Monthly patient enrollments by retraction strategy (Jan 2022–Mar 2024).***conventional retraction (light gray) and FLDM-NRR+IONM (dark gray). Vertical dotted line = institutional FLDM-NRR introduction (Jan 2023). Both strategies used concurrently (Jan 2023–Mar 2024).*
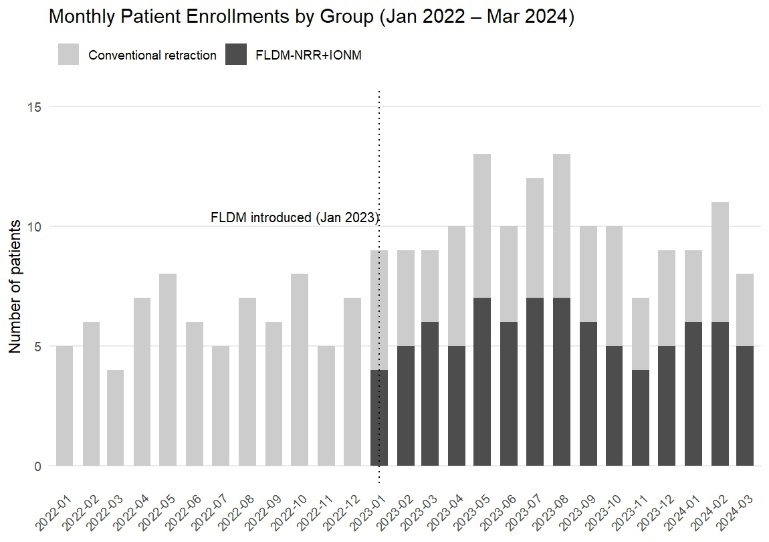


**Data Extraction and Standardization Procedures**

Data extraction was performed by independent research assistants who were blinded to group allocations (FLDM-NRR or conventional retraction) to prevent bias. Only essential information for analysis was provided, minimizing observer bias.

Inter-rater agreement was assessed using Cohen's Kappa statistic, showing high consistency with Kappa values of 0.85 for pain and 0.82 for neurological evaluations, indicating excellent reliability.

Postoperative visit schedules and electronic medical record (EMR) templates were standardized across both groups, ensuring consistent data recording. Pain and neurological assessments followed a uniform protocol using the Japanese Orthopedic Association (JOA) score, Oswestry Disability Index (ODI), and Visual Analog Scale (VAS) for pain, applied at all follow-up visits (1 week, 1 month, 3 months, and 12 months) to minimize detection bias.

Retrospective data extraction employed dual independent extraction with cross-verification, keeping key variable missing rates <5%. Of 312 initially assessed medical records, 50 were excluded based on inclusion/exclusion criteria, leaving 262 patients eligible for 12-month follow-up. Among these, 28 were lost to follow-up, and 234 completed follow-ups, yielding an 89.3% (234/262) 12-month follow-up data completeness rate—well above the acceptable threshold and far below the 15% clinical follow-up loss threshold and meeting retrospective cohort study data completeness requirements.

**Surgical Technique and Monitoring**

All surgeries were conducted by the same spinal surgery team using a standardized posterior midline approach. The PLIF procedure involved pedicle screw insertion, laminectomy, and decompression of the L5 nerve root. Nerve root retraction was performed at the shoulder region of the L5 nerve root, -proximal to the dural sleeve transition zone, where the nerve root exits the thecal sac.

**FLDM-NRR group:** The FLDM-NRR was used with the associated technical specifications: (1) The device based on the inherent ratchet constant-force mechanism of a micrometer; Following assembly, a force of 3.5 N (±0.5 N) was measured using external spectrometry and weight testing; (2) Displacement measuring precision: ±0.1 mm; (3) The nerve root displacement distance was determined by the numerical difference value between initial and final retraction positions of the nerve root as displayed on the device; (4) Preoperative calibration of the equipment was conducted to guarantee measurement precision. Subsequently, lateral bone decompression was performed once the nerve root retraction threshold was reached, so as to expand the surgical field (Figure 2-4).

**Figure 2. Design Schematic of the FLDM-NRR Device**

**
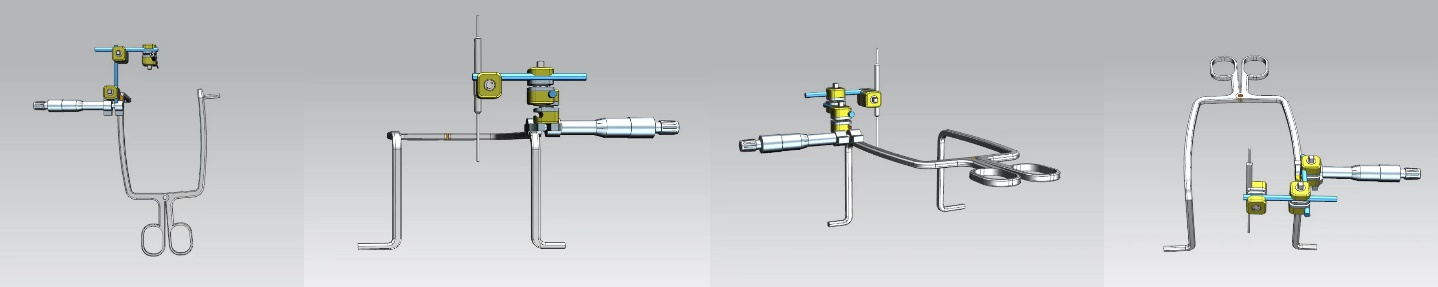
**

**Figure 3. Pre-clinical Validation: In Vitro Spectrometer Calibration and Cadaveric Testing**

**
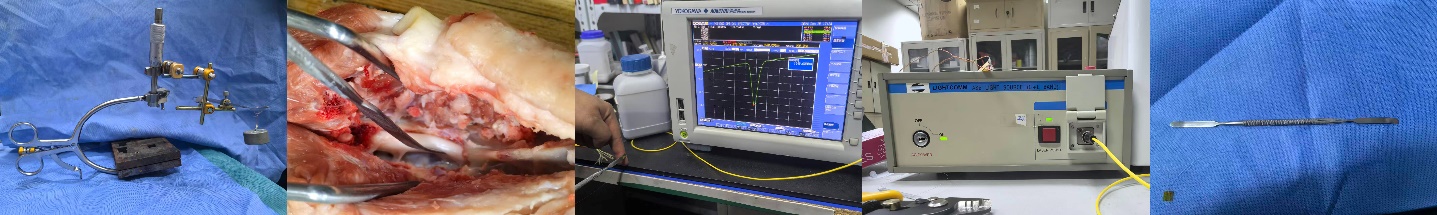
**

**Figure 4. Intraoperative Application of the FLDM-NRR System**

**
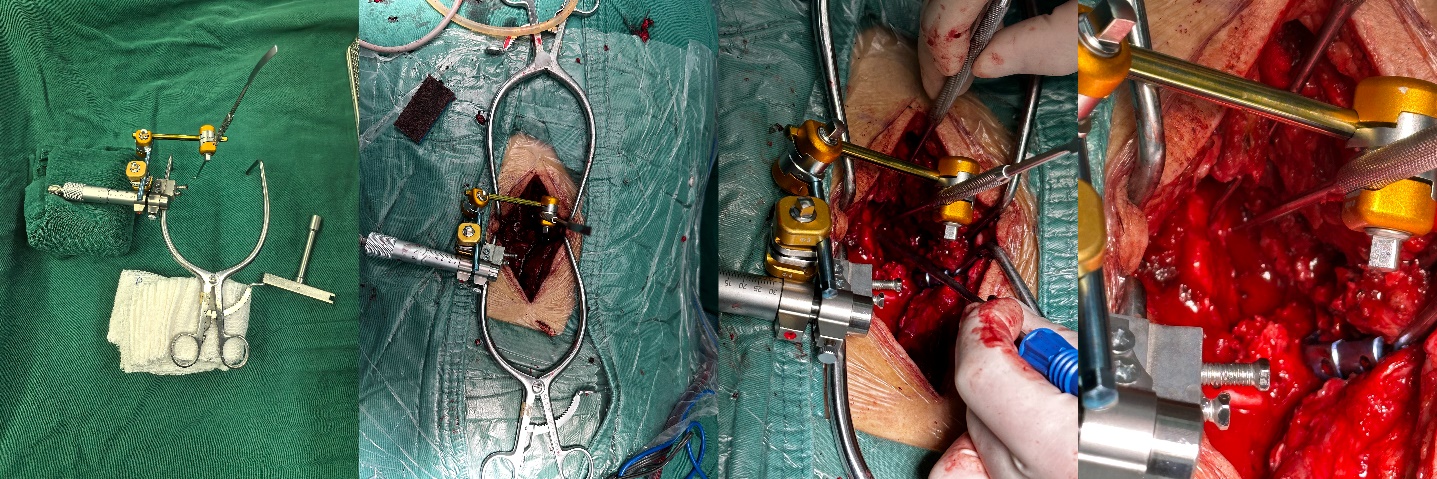
**

**Conventional group:** Standard nerve root retractors were used, with the force and distance of retraction reliant on the surgeon's clinical expertise and tactile input.

**Intraoperative Neurophysiological Monitoring**

The FLDM-NRR group received multimodal IONM using the Nicolet Endeavor CR system in accordance with the recommendations of the American Society of Neurophysiological Monitoring (ASNM). Criteria for alerts included: (1) A drop of ≥50% in the amplitude of somatosensory evoked potentials (SSEPs) or a lengthening of ≥10% in latency; (2) A loss of the amplitude of motor evoked potentials (MEPs) or a sustained decline of ≥80%in MEP amplitude; (3) Burst activity in Electromyography (EMG) of ≥5 bursts per second or the presence of neurotronic potentials.

Upon the activation of alarms, nerve root retraction was promptly halted, and the surgical was adjusted only once neurophysiological signals had returned to baseline values. All monitoring data were systematically documented and analyzed by experienced neurophysiological technicians in order to ensure inter-operator consistency (Figure 5).

**Figure 5.** Intraoperative Neurophysiological Monitoring


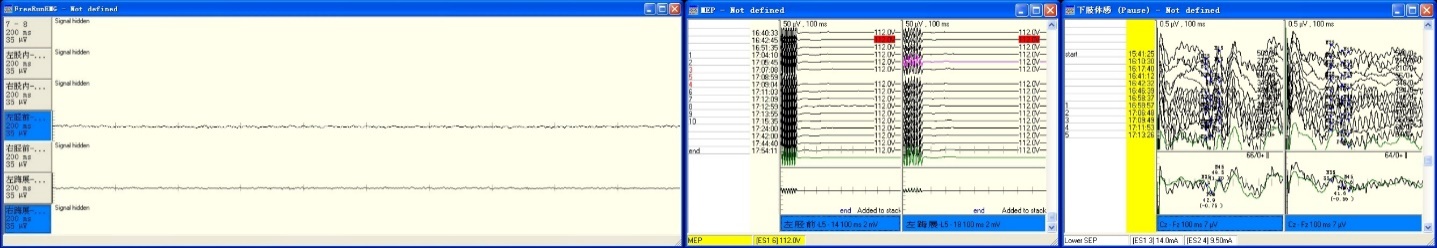


**Outcome Assessment**

**The** primary Outcomes were the frequency of new-onset L5 radiculitis during the three months following surgery and the rate of persisting neurological impairment at the one-year follow-up assessment. L5 radiculitis is characterized by any of the following findings relative to the preoperative baseline: a decrease in muscle strength of ≥1 grade in L5-innervated muscle groups (the Medical Research Council [MRC] scale), the emergence of sensory loss or reduced sensation in the L5 dermatome, or the onset or exacerbation of pain in the anatomical distribution of the L5 nerve root. Neurological deterioration was characterized by any newly developed or advancing motor dysfunction, sensory decline, a JOA score reduction of ≥3 points from baseline, or the emergence or exacerbation of neurogenic claudication relative to the preoperative state.

**The** Secondary Outcomes included functional assessment metrics, specifically the Japanese Orthopedic Association (JOA) score (0-29 points), Oswestry Disability Index (ODI, 0-100%), and Visual Analog Scale (VAS) scores for leg and back discomfort (0-10 points). Perioperative parameters included (total operative duration, interbody fusion procedure duration, intraoperative blood loss volume, postoperative drainage volume, duration of hospital stay, and postoperative disc height), device performance metrics included (nerve root retraction distance and rate of necessity for lateral bone decompression), and IONM efficacy parameters included (incidence rate of alerts, signal recovery status, and sensitivity and specificity for predicting postoperative complications)

**Statistical Analysis**

Continuous variables were presented as mean ± standard deviation (SD) or median (interquartile range [IQR]) and analyzed using Student's t-test or the Mann-Whitney U test. Categorical variables were summarized as n (%and analyzed using the chi-square**(χ²)** test or Fisher's exact test. Effect sizes were reported as relative risk (RR), or mean difference (MD),with 95% confidence intervals (CL). Sensitivity analyses included full case analysis, multivariable logistic regression with covariate adjustment (to account for confounding variables), surgeon-stratified fixed-effects models, monthly calendar coding, and secular-trend control (i.e., calendar quarter + surgeon random intercept). The primary endpoint (3-month L5 radiculitis incidence) was analyzed using multivariable logistic regression under hierarchical testing (α = 0.05); secondary time/functional endpoints were interpreted only if the primary endpoint was statistically significant, with time-trend interaction assessed using the Cochran–Armitage test. Longitudinal trajectories of ODI, JOA, and VAS scores were evaluated using a mixed-effects model to assess the interaction between time and group (time × group). Analyses were performed using SPSS 26.0 (IBM Corp., Armonk, NY, USA) and R 4.5.1, including adjustment for confounding variables, stratified analysis by operating surgeon, and application of two-tailed tests; statistical significance was set at P < 0.05, unless otherwise specified.

**Results**

**Baseline Characteristics**

During the study period, 234 patients were enrolled comprising 96 patients in the FLDM-NRR with IONM group and 138 patients in the conventional group. Patient allocation was based on the objective availability of the FLDM-NRR device following its institutional introduction in January 2023, this availability was determined by sterilization cycles and maintenance schedules with no subjective selection criteria applied.

Baseline characteristics demonstrated excellent comparability between the two groups (Table 1). No significant differences were observed in demographics, clinical presentation, or preoperative functional assessment (all P > 0.05).

**Table 1.** Baseline demographic and clinical characteristics

| **Characteristic** | **FLDM-NRR Group (n=96)** | **Conventional Group (n=138)** | **P Value** |
| --- | --- | --- | --- |
| *Demographics* | | | |
| Age, years | 59.87 ± 7.23 | 61.15 ± 8.34 | 0.243 |
| Male sex, n (%) | 50 (52.08) | 71 (51.45) | 0.928 |
| BMI, kg/m² | 26.93 ± 2.84 | 27.38 ± 3.12 | 0.314 |
| *Clinical Presentation* | | | |
| Symptom duration, months | 27.64 ± 15.27 | 30.83 ± 16.45 | 0.182 |
| Neurogenic claudication, n (%) | 78 (81.25) | 115 (83.33) | 0.694 |
| Radicular pain, n (%) | 72 (75.00) | 108 (78.26) | 0.584 |
| *Comorbidities* | | | |
| Type 2 diabetes, n (%) | 16 (16.67) | 27 (19.57) | 0.546 |
| Hypertension, n (%) | 34 (35.42) | 52 (37.68) | 0.713 |
| Current/former smoker, n (%) | 15 (15.63) | 25 (18.12) | 0.638 |
| *Functional Assessments* | | | |
| JOA score (0-29) | 11.52 ± 2.83 | 11.18 ± 2.91 | 0.407 |
| ODI score (%) | 57.84 ± 8.37 | 59.86 ± 8.94 | 0.273 |
| VAS back pain score | 6.98 ± 1.24 | 7.42 ± 1.31 | 0.152 |
| VAS leg pain score | 7.73 ± 1.29 | 7.51 ± 1.38 | 0.379 |
| *Radiological Features* | | | |
| Dural sac area, mm²* | 80.15 ± 12.53 | 82.29 ± 13.84 | 0.327 |
| Preoperative disc height, mm† | 6.18 ± 0.87 | 6.13 ± 0.82 | 0.689 |
| Severe stenosis (≤75 mm²), n (%) | 51 (53.13) | 64 (46.38) | 0.297 |
| Grade III-IV facet degeneration‡, n (%) | 72 (75.00) | 108 (78.26) | 0.563 |

*Data presented as mean ± SD or n (%).*

**Measured on axial T2-weighted MRI at most stenotic level.*

*†Measured on preoperative lateral radiographs as the average of anterior, middle, and posterior disc heights at L4-L5 level.*

*‡Graded according to Weishaupt classification for facet joint degeneration.*

*P values calculated using independent t-tests for continuous variables and χ² test for categorical variables.*

**Primary Outcomes**

In the unadjusted analysis, the FLDM-NRR group significantly reduced L5 radiculitis incidence up to three months after surgery (7/96 [7.29%] vs 25/138 [18.12%], RR 0.40, 95% CI 0.18 - 0.91, P=0.028; absolute risk reduction 10.83%, number needed to treat 9, 95 % CI 2.6–19.1 % / 5.2–39.1 %). (table 2).

Time-stratified analysis showed the most profound effect in the first week after surgery 3.13% vs 13.04%, RR 0.24, P=0.020, which gradually waned during the subsequent periods. Cochran–Armitage trend-interaction test revealed a significantly steeper decline of L5 radiculitis over time in the FLDM-NRR group (P=0.032, Table 2).The benefits were not evident at a one-year follow-up, with no corresponding absolute benefit for persistent neurological deficit (ARR 4.1 %, 95 % CI −2.0 % to 10.2 %, P=0.200; RR 0.43, 95 % CI 0.12–1.56)..

After adjustment for all pre-specified covariates, the protective effect of FLDM-NRR remained significant (adjusted OR 0.31, 95 % CI 0.12–0.78, P=0.017; Supplementary_Tables_Sm1). Robustness was confirmed across five sensitivity models (RR 0.24–0.36, all P≤0.022; Sy), including surgeon-fixed-effect (aOR 0.30, P=0.015, Sm2a/2b) and monthly calendar coding (RR 0.24, P=0.006), with no inter-surgeon heterogeneity (χ²=0.02, P=0.99, Sm2b).

**Table 2.** Primary outcomes at 3-month and 1-year follow-up

| **Primary Endpoint** | **FLDM-NRR Group (n=96)** | **Conventional Group (n=138)** | **Relative Risk (95% CI)** | **P Value** |
| --- | --- | --- | --- | --- |
| **New-onset L5 Radiculitis at 3 Months** | | | | |
| Overall incidence | 7/96 (7.29) | 25/138 (18.12) | 0.40 (0.18 to 0.91) | **0.028** |
| **Adjusted OR (95 % CI)** | - | - | 0.31 (0.12–0.78) | **0.017** |
| **Severity Stratification** | | | | |
| Grade I (Mild) | 5/96 (5.21) | 16/138 (11.59) | 0.45 (0.17 to 1.20) | 0.108 |
| Grade II (Moderate) | 2/96 (2.08) | 7/138 (5.07) | 0.41 (0.09 to 1.92) | 0.259 |
| Grade III (Severe) | 0/96 (0.00) | 2/138 (1.45) | -- | 0.535 |
| **Trend-interaction test** | - | - | - | **0.032** |
| **Symptom Categories** | | | | |
| Pain aggravation (VAS ≥2↑) * | 4/96 (4.17) | 14/138 (10.14) | 0.41 (0.14 to 1.23) | 0.112 |
| Sensory deterioration | 2/96 (2.08) | 8/138 (5.80) | 0.36 (0.08 to 1.66) | 0.189 |
| Motor function decline (≥1 MRC↓) † | 1/96 (1.04) | 5/138 (3.62) | 0.29 (0.03 to 2.44) | 0.252 |
| **Time-stratified Analysis** | | | | |
| ≤1 week postoperative | 3/96 (3.13) | 18/138 (13.04) | 0.24 (0.07 to 0.80) | **0.020** |
| 1 week to 1 month | 3/96 (3.13) | 5/138 (3.62) | 0.86 (0.21 to 3.53) | 0.836 |
| 1-3 months | 1/96 (1.04) | 2/138 (1.45) | 0.72 (0.07 to 7.79) | 0.786 |
| **Recovery Pattern at 3 Months** | | | | |
| Complete recovery | 6/7 (85.71) | 16/25 (64.00) | 1.34 (0.81 to 2.21) | 0.252 |
| Partial recovery | 1/7 (14.29) | 7/25 (28.00) | 0.51 (0.07 to 3.60) | 0.493 |
| No improvement | 0/7 (0.00) | 2/25 (8.00) | -- | 1.000 |
| **Persistent Neurological Deterioration at 1 Year** | | | | |
| Any persistent deterioration | 3/96 (3.13) | 10/138 (7.25) | 0.43 (0.12 to 1.56) | 0.200 |
| Persistent motor weakness | 1/96 (1.04) | 4/138 (2.90) | 0.36 (0.04 to 3.18) | 0.361 |
| Persistent sensory deficit | 1/96 (1.04) | 4/138 (2.90) | 0.36 (0.04 to 3.18) | 0.361 |
| Functional deterioration (JOA decline ≥3) ‡ | 0/96 (0.00) | 2/138 (1.45) | -- | 0.535 |
| Persistent/worsened claudication | 1/96 (1.04) | 2/138 (1.45) | 0.72 (0.07 to 7.79) | 0.786 |
| *Long-term Recovery Analysis* | | | | |
| Complete neurological recovery at 1 year | 93/96 (96.88) | 128/138 (92.75) | 1.04 (0.99 to 1.10) | 0.111 |
| Residual symptoms affecting daily life | 2/96 (2.08) | 8/138 (5.80) | 0.36 (0.08 to 1.66) | 0.189 |

***Recovery Pattern Criteria at 3 Months:***

*Complete recovery: Complete resolution of L5 radiculitis symptoms, return to baseline neurological function*

*Partial recovery: ≥50% improvement in symptom severity but residual symptoms persist*

*No improvement: <50% improvement in symptoms or symptom progression*

***Grade for New-onset L5 Radiculitis:***

*Grade I (Mild): Single symptom, minimal functional impact*

*Grade II (Moderate): Multiple symptoms or moderate functional limitation*

*Grade III (Severe): Significant motor deficit with major functional impairment*

*Data presented as events/total available patients (%). *VAS increase ≥2 points from baseline. †Medical Research Council muscle strength scale. ‡Japanese Orthopedic Association score decline. P < 0.05 (two-tailed tests). Fisher's exact test for sparse data.*

*Adjusted OR derived from multivariable logistic regression including age, sex, BMI, baseline pain/ODI/JOA, diabetes, smoking, Lee grade, dural sac area, surgeon (random intercept).*

*Secondary time-stratified comparisons interpreted under hierarchical testing; trend-interaction test represents the overall time group interaction*

**Secondary Outcomes**

Functional evaluations demonstrated progressive improvements that favored the FLDM-NRR group (Table 3). Early benefits were evident in VAS leg pain scores at one week (4.83±1.94 vs 5.68±2.12 points, P<0.001). At the one-year follow-up, the FLDM-NRR group showed superior ODI scores (18.43±7.85% vs 21.17±8.42%, P=0.018) and sustained reduction in leg and back pain.

MCID analysis showed the FLDM-NRR group had a significantly higher proportion of patients reaching the MCID for VAS-leg pain (95.8% vs 93.5%; P = 0.028) and a non-significant trend toward a higher proportion for ODI (87.5% vs 78.3%; P = 0.070).

Mixed-effects models revealed significant group effects favoring the FLDM-NRR group for VAS-leg pain (P < 0.001) and ODI (P < 0.001) across all time points; with larger between-group differences observed at 1 week postoperatively. JOA scores were consistently higher in the FLDM-NRR group (group effect: P < 0.001), but there was a minimal time×group interaction (P > 0.05)（**Supplementary_Tables_Sn1~3**）.

**Table 3.** Functional outcomes at baseline and follow-up visits

| **Assessment** | **FLDM-NRR Group (n=96)** | **Conventional Group (n=138)** | **Mean Difference (95% CI)** | **P Value** |
| --- | --- | --- | --- | --- |
| *JOA Score (0-29 points)* | | | | |
| Baseline | 11.52 ± 2.83 | 11.18 ± 2.91 | 0.34 (-0.43 to 1.11) | 0.410 |
| 1 week postoperative | 14.23 ± 3.14 | 13.87 ± 3.32 | 0.36 (-0.58 to 1.30) | 0.520 |
| 3 months postoperative | 21.14 ± 3.67 | 20.58 ± 4.12 | 0.56 (-0.47 to 1.59) | 0.340 |
| 1 year postoperative | 24.87 ± 3.24 | 24.03 ± 3.65 | 0.84 (-0.09 to 1.77) | 0.078 |
| *ODI Score (%)* | | | | |
| Baseline | 57.84 ± 8.37 | 59.86 ± 8.94 | -2.02 (-5.31 to 1.27) | 0.270 |
| 1 week postoperative | 49.18 ± 9.12 | 50.79 ± 9.64 | -1.61 (-4.23 to 1.01) | 0.230 |
| 3 months postoperative | 27.76 ± 8.93 | 29.42 ± 9.87 | -1.66 (-4.18 to 0.86) | 0.210 |
| 1 year postoperative | 18.43 ± 7.85 | 21.17 ± 8.42 | -2.74 (-5.06 to -0.42) | **0.018** |
| *VAS Leg Pain Score* (0-10)* | | | | |
| Baseline | 7.73 ± 1.29 | 7.51 ± 1.38 | 0.22 (-0.27 to 0.71) | 0.380 |
| 1 week postoperative | 4.83 ± 1.94 | 5.68 ± 2.12 | -0.85 (-1.37 to -0.33) | **<0.001** |
| 1 month postoperative | 3.17 ± 1.73 | 4.08 ± 1.89 | -0.91 (-1.38 to -0.44) | **<0.001** |
| 3 months postoperative | 2.14 ± 1.42 | 2.57 ± 1.63 | -0.43 (-0.87 to 0.01) | **0.025** |
| 1 year postoperative | 1.18 ± 1.04 | 1.53 ± 1.17 | -0.35 (-0.64 to -0.06) | 0.067 |
| *VAS Back Pain Score† (0-10)* | | | | |
| Baseline | 6.98 ± 1.24 | 7.42 ± 1.31 | -0.44 (-0.82 to -0.06) | 0.150 |
| 1 week postoperative | 4.87 ± 1.63 | 5.16 ± 1.82 | -0.29 (-0.74 to 0.16) | 0.140 |
| 3 months postoperative | 2.23 ± 1.34 | 2.58 ± 1.52 | -0.35 (-0.73 to 0.03) | 0.057 |
| 1 year postoperative | 1.27 ± 0.92 | 1.59 ± 1.08 | -0.32 (-0.57 to -0.07) | 0.081 |
| ODI score (MCID > 12%) | 84/96 (87.50%) | 108/138 (78.26%) |  | 0.070 |
| VAS leg score (MCID > 2) | 92/96 (95.83%) | 119/138 (93.48%) |  | 0.028 |
| VAS Back score (MCID > 2 | 85/96 (88.54%) | 115/138 (83.33%) |  | 0.356 |

*Data presented as mean ± SD. MD, mean difference; CI, confidence interval.*

*JOA, Japanese Orthopedic Association (range 0-29, higher scores indicate better function); ODI, Oswestry Disability Index (range 0-100%, lower scores indicate better function).*

**Leg pain including radicular pain and neurogenic claudication symptoms. †Low back pain intensity.*

*P values calculated using independent t-tests.*

*Secondary endpoints (JOA/ODI/VAS), interpreted via hierarchical testing (primary endpoint P=0.017), no separate P-adjustment.*

**Device Performance and IONM Efficacy**

The FLDM-NRR system maintained 100% adherence to preset force limits (≤3.50N) with zero mechanical failures across 96 procedures (Table 4). The mean nerve root retraction distance was 5.84±1.17 mm. Lateral bone decompression was required in 19.79% of cases due to conditions where the force-limited was reached.

**Table 4.** FLDM-NRR device performance and surgical outcomes (n=96)

| **Parameter/Outcome** | **Value** | **95% CI** |
| --- | --- | --- |
| *Device Performance* | | |
| Force limitation threshold, N | 3.50 | -- |
| Force threshold violations, n (%) | 0 (0.00) | (0.00 to 3.79) |
| Mean nerve root displacement, mm | 5.84 ± 1.17 | (5.60 to 6.08) |
| *Surgical Adaptations* | | |
| Lateral bone decompression required*, n (%) | 19 (19.79) | (12.25 to 29.22) |
| Papaverine irrigation applied†, n (%) | 3 (3.13) | (0.64 to 8.83) |
| Total intraoperative adjustments | 18 | -- |
| *Safety Outcomes* | | |
| Device-related adverse events, n (%) | 0 (0.00) | (0.00 to 3.79) |

*Data presented as n (%), mean ± SD with 95% CI, or absolute count.*

**When force limit insufficient for adequate surgical exposure.*

*†Applied for nerve root tension signs or vasospasm prevention.*

IONM alerts occurred in 9.38% of cases, with signal recovery achieved in 77.78% of these alert cases within 30 minutes (Table 5). IONM demonstrated high diagnostic accuracy for predicting postoperative complications, with a sensitivity of 85.71% and a specificity of 96.63%,

**Table 5.** Intraoperative neurophysiological monitoring alerts and diagnostic performance (n=96)

| **Monitoring Parameter** | **Cases (n)** | **Rate (%)** | **95% CI** |
| --- | --- | --- | --- |
| *Alert Events* | | | |
| Any IONM alert* | 9 | 9.38 | (4.33 to 17.12) |
| SSEP abnormalities† | 3 | 3.13 | (0.64 to 8.83) |
| MEP abnormalities‡ | 4 | 4.17 | (1.14 to 10.31) |
| Free-running EMG alerts§ | 5 | 5.21 | (1.70 to 11.73) |
| Multiple alert types | 2 | 2.08 | (0.25 to 7.33) |
| *Alert Recovery* | | | |
| Complete signal recovery¶ | 7 | 77.78 | (40.02 to 97.19) |
| Partial signal recovery | 2 | 22.22 | (2.81 to 59.98) |
| Persistent abnormalities | 0 | 0.00 | (0.00 to 33.63) |
| *Diagnostic Accuracy* | | | |
| Sensitivity | -- | 85.71 | (42.13 to 99.64) |
| Specificity | -- | 96.63 | (90.36 to 99.30) |
| Positive predictive value | -- | 66.67 | (29.93 to 92.51) |
| Negative predictive value | -- | 97.70 | (91.92 to 99.72) |

*Data presented as n (%) with exact 95% confidence intervals calculated using Clopper-Pearson method.*

**Any SSEP, MEP, or EMG change meeting predetermined ASNM criteria.*

*†≥50% amplitude decrease or ≥10% latency prolongation in tibial/peroneal SSEP.*

*‡≥80% amplitude loss or complete signal loss in lower extremity MEPs.*

*§Burst activity (≥5/second) or neurotronic discharges.*

*¶Signal return to ≥90% baseline within 30 minutes. Recovery times: complete 6.8 ± 3.2 min; partial 15.2 ± 4.1 min.*

*Diagnostic accuracy calculated against postoperative L5 radiculitis as reference standard.*

**Perioperative Safety**

No significant differences were observed between the two groups in operative time (132.48±18.73 vs 129.85±21.27 minutes, P=0.462), intraoperative blood loss (235.64±58.31 vs 248.69±62.47 mL, P=0.241), or duration of hospital stay (11.82±1.19 vs 12.27±1.34 days, P=0.292), thus confirming that the FLDM-NRR technology did not compromise surgical efficiency (Table 6).

**Table 6**. Surgical Parameters and Perioperative Outcomes

| Parameter | FLDM-NRR Group (n=96) | Conventional Group (n=138) | Effect Size (95% CI) | P Value |
| --- | --- | --- | --- | --- |
| Operative time (min) | 132.48 ± 18.73 | 129.85 ± 21.27 | MD 2.63 (-4.41 to 9.67) | 0.462 |
| Interbody fusion time (min)* | 11.24 ± 1.82 | 10.79 ± 2.08 | MD 0.45 (-0.53 to 1.43) | 0.316 |
| Blood loss (mL) | 235.64 ± 58.31 | 248.69 ± 62.47 | MD -13.05 (-34.82 to 8.72) | 0.241 |
| Postoperative drainage (mL) | 309.87 ± 45.23 | 315.14 ± 48.62 | MD -5.27 (-16.91 to 6.37) | 0.398 |
| Length of stay (days) | 11.82 ± 1.19 | 12.27 ± 1.34 | MD -0.45 (-1.18 to 0.28) | 0.292 |
| Postoperative disc height (mm)† | 8.93 ± 0.79 | 8.81 ± 0.72 | MD 0.12 (-0.31 to 0.55) | 0.621 |

*Data presented as mean ± SD. MD, mean difference.*

**Duration from annulus fibrosus incision to cage placement.*

*†Measured on lateral radiographs at 1-week follow-up.*

*P values calculated using independent t-tests*.

**Discussion**

This retrospective study indicates that the FLDM-NRR system combined with IONM significantly reduces the incidence of L5 radiculitis after single-level PLIF from 18% to 7%( absolute risk reduction [ARR]: 10.8%; number needed to treat [NNT]: 9; 95% confidence interval [CI]: 5–39). The neuroprotective effect was particularly significant in the first postoperative week(Cochran–Armitage trend interaction: P = 0.032), with this benefit remaining consistent after multivariable adjustment for calendar time, surgeon variability, and all pre-specified covariates—indicating that early mechanical nerve root trauma is the critical trigger for subsequent neuroinflammatory cascades. In our clinical experience, this approach has demonstrated significant utility in addressing one of the most technically challenging aspects of PLIF surgery—striking an optimal balance between adequate neural decompression and the prevention of iatrogenic injury—by providing a quantitative solution.

The identified neuroprotective effects rely on two core principles: meticulous mechanical force regulation and the disruption of pathogenic neuroinflammatory cascades. Biomechanical research indicates that the acceptable limits for lumbar nerve root traction damage consist of individual traction forces below 4.1±0.45 N and cumulative traction loads (force × time) under 42.89±2.96N·min [13]. Experimental validation verifies that traction forces in the 1-6N range are within acceptable tolerance limits, with adjacent ligamentous structures providing good protective support [18]. Prior technical validation using fiber-optic sensors in cadaveric studies determined the necessary traction force range of 3.0-4.5N for secure cage placement. This research determined the 3.5N threshold to optimize neuroprotection while maintaining surgical practicality, based on both established biomechanical concepts and our clinical surgical expertise. The pathophysiological analysis of the present study indicates that the timing of neuroprotective effects underscores the paramount significance of early prevention of mechanical injury: the most substantial risk reduction occurred within the initial postoperative week (76%), subsequently declining over time, thereby suggesting that averting initial mechanical trauma effectively disrupts neuroinflammatory cascades, including microglial activation and the release of pro-inflammatory cytokine release [19-22].

The FLDM-NRR technology signifies a transition from subjective surgical experience to objective mechanical measurement, in contrast to traditional nerve root retraction approaches. Conventional approaches rely mostly on the surgeon's clinical expertise and tactile feedback, lacking standardized criteria and thereby leading to considerable inter-operator variability and procedural unpredictability. The FLDM-NRR technique establishes consistent, measurable neuroprotective criteria via real-time mechanical force monitoring and displacement feedback mechanisms.

The relatively elevated incidence of radiculitis in our control group (18.1%) reflects the adoption of more rigorous diagnostic criteria, which include nuanced sensory alterations, pain characteristics, and variations in pain distribution patterns, in contrast to earlier studies that focused primarily on overt motor deficits. Using these same diagnostic criteria, FLDM-NRR group achieved a radiculitis rate of 7.3%, which is lower than the 7.8% nerve root injury rate reported by Okuda et al. [23] and pooled complication rates (>) 5.2% in recent systematic reviews [24-25], thereby highlighting distinct technological advantages.

The effectiveness study of IONM demonstrated that our integrated monitoring approach achieved 85.7% sensitivity and 96.6% specificity. In contrast to previous systematic evaluations indicating a multimodal monitoring sensitivity of 83.5% and specificity of 93.8% [26-28]. Our analysis indicated slightly enhanced sensitivity and improved specificity, implying benefits in minimizing false-positive warnings and preventing unwarranted surgical procedures. The one-year rates of persistent neurological impairment were 3.1% in the FLDM-NRR group compared to 7.2% in the conventional group (P=0.200). Despite the lack of statistical significance, the 57% relative risk (RR) reduction has significant clinical implications. From a pathophysiological standpoint, enduring neurological impairment is intricately linked to prolonged microglial activation and chronic neuroinflammation [29-30]. Mechanical nerve damage induces microglial activation from a resting state to pro-inflammatory phenotypes, resulting in the release of pro-inflammatory cytokines such as TNF-α and IL-1β, thereby establishing a neuroinflammatory feedback loop [20]. Subsequent to peripheral nerve damage, spinal microglial cells exhibit considerable proliferation and phenotypic alterations in both the dorsal and ventral horn regions, participating in intricate interactions with diverse spinal cellular constituents [31]. The significant decrease in early postoperative radiculitis seen in this study clearly demonstrates the efficacy of FLDM-NRR technology in disrupting pathogenic cascades via the management of initial mechanical damage. In comparison to the current PLIF research, the persistent neurological deterioration rate of our conventional group (7.2%) is within acceptable limits, since literature indicates PLIF neurological damage rates of 9-16% [32 )]. Conversely, complication rates in our FLDM-NRR cohort were consistently lower than those reported in typical PLIF studies.

**Study Limitations**

This study is limited by its retrospective, single-Centre design and device allocation based on sterilization cycles rather than randomization; however, concurrent enrolment, calendar-quarter adjustment, and surgeon-stratified sensitivity analyses yielded consistent effect estimates. Early cases during device introduction (January 2023) may reflect surgeon learning curves [(4)], but calendar-time modelling mitigates this bias. The 10.7 % loss-to-follow-up rate and best-/worst-case bounds (RR 0.39–0.50) confirm robustness. The micrometer-based retractor has a fixed 3.5 N threshold (±0.5 N bench deviation) and measures transmitted rather than tissue-contact force; adjustable or sensor-integrated retractor designs are under development. Multicenter, prospective, randomized trials with ≥5-year follow-up are needed to confirm long-term neurological durability and to benchmark FLDM-NRR against other standardized retraction technologies.

**Future Directions**

Next, multicenter, prospective, randomized trials with ≥5-year neurological follow-up should benchmark FLDM-NRR against other standardized retraction technologies. Ultimately, integrating contact-pressure sensors and AI-driven, patient-specific force thresholds will convert the current transmitted-force system into genuine tissue-feedback instrumentation, enabling robotic-platform deployment and segment-specific optimization.

**Conclusions**

FLDM-NRR combined with IONM quantitatively reduces the incidence of early L5 radiculitis after PLIF (ARR: 10.8 %, NNT: 9, 95 % CI: 2.6–19.1 %); notably, the proportion of patients achieving the MCID was superior in the FLDM-NRR group for VAS-leg pain scores. However, this approach confers no absolute benefit on persistent neurological deficit at the 1-year follow-up. Next, multicenter, prospective, randomized trials with ≥5-year neurological follow-up should benchmark this force-limited strategy against other standardized retraction technologies. Ultimately, integrating contact-pressure sensors and AI-driven, patient-specific force thresholds will convert transmitted-force instrumentation into genuine tissue-feedback systems—enabling robotic-platform deployment and segment-specific optimization for spinal surgery.

**Funding**

This work was supported by the Natural Science Foundation of Tianjin (Grant No. 23JCZDJC00830), Sponsored by Tianjin Health Research Project (GrantNo.TJWJ2024QN057), and Hainan Provincial Natural Science Foundation Project (Grant No. 824QN379).

**References**

[1] Jensen RK, Jensen TS, Koes B, Hartvigsen J. Prevalence of lumbar spinal stenosis in general and clinical populations: a systematic review and meta-analysis. Eur Spine J. 2020;29(9):2143-63.

[2] Lurie J, Tomkins-Lane C. Management of lumbar spinal stenosis. JAMA. 2022;327(17):1688-99.

[3] Zaina F, Tomkins-Lane C, Carragee E, Negrini S. Surgical versus non-surgical treatment for lumbar spinal stenosis. Cochrane Database Syst Rev. 2016;1:CD010264.

[4] de Kunder SL, van Kuijk SMJ, Rijkers K, Caelers IJMH, van Hemert WLW, de Bie RA, et al. Transforaminal versus posterior lumbar interbody fusion for symptomatic single-level spondylolisthesis (LIFT): a multicentre controlled, patient blinded, randomised non-inferiority trial. Lancet Reg Health Eur. 2024;40:100881.

[5] Lan T, Hu S, Zhang Y, Zheng Y, Zhang R, Shen Z, et al. Comparison between posterior lumbar interbody fusion and transforaminal lumbar interbody fusion for the treatment of lumbar degenerative diseases: a systematic review and meta-analysis. World Neurosurg. 2018;112:86-93.

[6] Kaťuch V, Grega R, Knorovsky K, Banoci J, Katuchova J, Šašala M, et al. Comparison between posterior lumbar interbody fusion and transforaminal lumbar interbody fusion in the management of lumbar spondylolisthesis. Bratisl Lek Listy. 2021;122(9):653-6.

[7] Dowlati E, Alexander H, Voyadzis J. Vulnerability of the L5 nerve root during anterior lumbar interbody fusion at L5-S1: case series and review of the literature. Neurosurg Focus. 2020;49(3):E7.

[8] Liu J, Deng H, Long X, Chen X, Xu R, Liu Z. A comparative study of perioperative complications between transforaminal versus posterior lumbar interbody fusion in degenerative lumbar spondylolisthesis. Eur Spine J. 2016;25:1575-80.

[9] Guzzi G, Ricciuti RA, Della Torre A, Lo Turco E, Lavano A, Longhini F, et al. Intraoperative neurophysiological monitoring in neurosurgery. J Clin Med. 2024;13(5):1234.

[10] Charalampidis A, Jiang F, Wilson JRF, Badhiwala J, Brodke D, Fehlings M. The use of intraoperative neurophysiological monitoring in spine surgery. Global Spine J. 2020;10(1):104-13.

[11] Cofano F, Zenga F, Mammi M, Altieri R, Marengo N, Ajello M, et al. Intraoperative neurophysiological monitoring during spinal surgery: technical review in open and minimally invasive approaches. Neurosurg Rev. 2019;42(2):371-89.

[12] Nunes R, Bersot C, Garritano J. Intraoperative neurophysiological monitoring in neuroanesthesia. Curr Opin Anaesthesiol. 2018;31(5):532-8.

[13] Guofeng B. Study of nerve root traction injury in lumbar interbody fusion from posterior route approach. Orthop J China. 2008;16(12):901-4.

[14] Matsui H, Kitagawa H, Kawaguchi Y, Tsuji H. Physiologic changes of nerve root during posterior lumbar discectomy. Spine. 1995;20(6):654-9.

[15] Winkelstein BA, DeLeo JA. Mechanical thresholds for initiation and persistence of pain following nerve root injury: mechanical and chemical contributions at injury. J Biomech Eng. 2004;126(2):258-63.

[16] Gerling M, Baker M, Stanton E, Chaladoff E, Buser Z. Nerve root retraction time during lumbar endoscopic discectomy: association with new onset radiculitis, a post-operative neurologic complication. Eur Spine J. 2023;32(11):3847-54.

[17] Lee GY, Lee JW, Choi HS, Oh KJ, Kang HS. A new grading system of lumbar central canal stenosis on MRI: an easy and reliable method. Skeletal Radiol. 2011;40(8):1033-9.

[18] Kraan GA, Smit TH, Hoogland PV, Snijders CJ. Lumbar extraforaminal ligaments act as a traction relief and prevent spinal nerve compression. Clin Biomech. 2010;25(1):10-5.

[19] Schenone A, Massucco S, Schenone C, Venturi C, Nozza P, Prada V, et al. Basic pathological mechanisms in peripheral nerve diseases. Int J Mol Sci. 2025;26(3):3377.

[20] Sommer C, Leinders M, Üçeyler N. Inflammation in the pathophysiology of neuropathic pain. Pain. 2018;159(Suppl 1):S95-S102.

[21] Coderre TJ, Katz J, Vaccarino AL, Melzack R. Contribution of central neuroplasticity to pathological pain: review of clinical and experimental evidence. Pain. 1993;52(3):259-85.

[22] Tsuda M. Microglia in the spinal cord and neuropathic pain. J Diabetes Investig. 2016;7(1):17-26.

[23] Okuda S, Miyauchi A, Oda T, Haku T, Yamamoto T, Iwasaki M. Surgical complications of posterior lumbar interbody fusion with total facetectomy in 251 patients. J Neurosurg Spine. 2006;4(4):304-9.

[24] Epstein NE. More nerve root injuries occur with minimally invasive lumbar surgery, especially extreme lateral interbody fusion: a review. Surg Neurol Int. 2016;7(Suppl 3):S83-95.

[25] Alvi MA, Kwon BK, Hejrati N, Tetreault L, Evaniew N, Skelly AC, et al. Accuracy of intraoperative neuromonitoring in the diagnosis of intraoperative neurological decline in the setting of spinal surgery: a systematic review and meta-analysis. Global Spine J. 2024;14(1 Suppl):105S-149S.

[26] Azad TD, Pendharkar AV, Nguyen V, Pan J, Connolly ID, Veeravagu A, et al. Diagnostic utility of intraoperative neurophysiological monitoring for intramedullary spinal cord tumors: systematic review and meta-analysis. Clin Spine Surg. 2018;31(3):112-9.

[27] Dauleac C, Boulogne S, Barrey C, Guyotat J, Jouanneau E, Mertens P, et al. Predictors of functional outcome after spinal cord surgery: relevance of intraoperative neurophysiological monitoring combined with preoperative neurophysiological and MRI assessments. Neurophysiol Clin. 2022;52(4):242-51.

[28] Baria E, Giordano F, Guerrini R, Caporalini C, Buccoliero A, Cicchi R, et al. Dysplasia and tumor discrimination in brain tissues by combined fluorescence, Raman, and diffuse reflectance spectroscopies. Biomed Opt Express. 2022;14(3):1256-75.

[29] Gąssowska-Dobrowolska M, Cieślik M, Czapski GA, Jęśko H. The role of neuroinflammation in neurodegenerative diseases: current understanding and future therapeutic targets. Front Aging Neurosci. 2024;15:1347987.

[30] Qin J, Ma Z, Chen X, Shu S. Microglia activation in central nervous system disorders: a review of recent mechanistic investigations and development efforts. Front Neurol. 2023;14:1103416.

[31] Rotterman TM, Nardelli P, Cope TC, Alvarez FJ. The role of microglia in neuroinflammation of the spinal cord after peripheral nerve injury. Cells. 2022;11(13):2083.

[32] Rajaee SS, Bae HW, Kanim LE, Delamarter RB. Posterior lumbar interbody fusion. Orthop Clin North Am. 2018;49(2):159-71

1. Wang W, Cui Y, Sun X, Zhang H, Yin W, Cui X, et al. Transforaminal posterior lumbar interbody fusion microscopic safe operating area: a three-dimensional model study based on computed tomography imaging. J Orthop Surg Res. 2024 June 8;19(1):342.

2. Bahir AW, Daxing W, Jiayu X, Bailian L, Shao G. Comparative efficacy and fusion outcomes of unilateral bi-portal endoscopic transforaminal lumbar interbody fusion versus minimally invasive transforaminal lumbar interbody fusion in treating single-segment degenerative lumbar spondylolisthesis with lumbar spinal stenosis: a two-year retrospective study. J Orthop Surg Res. 2024 Dec 19;19(1):835.

3. Arunakul R, Anumas S, Pattharanitima P, Susrivaraput C, Pholsawatchai W. Unilateral biportal endoscopic versus microscopic transforaminal lumbar interbody fusion for lumbar degenerative disease: a retrospective study. J Orthop Surg Res. 2024 June 1;19(1):326.

4. Guo W, Ye J, Li T, Yu Y, Fan X. Evaluation of the learning curve and complications in unilateral biportal endoscopic transforaminal lumbar interbody fusion: cumulative sum analysis and risk-adjusted cumulative sum analysis. J Orthop Surg Res. 2024 Mar 21;19(1):194.
